# Au/GQD hybrid interfaces: signal-transduction principles and evidence for metal-ion sensing

**DOI:** 10.1039/d6ra10016c

**Published:** 2026-11-04

**Authors:** Asokan Vasudevan, Qusay Abdulsattar Mohammed, Seif Al Bustanji, Vikram V. Patel, B. Rajalingam, Harvinder Singh Sohal, I. B. Sapaev, Suleiman Ibrahim Mohammad, Sahar Bayatinia

**Affiliations:** a Faculty of Business and Communications, INTI International University, Persiaran Perdana BBN Putra Nilai 71800 Nilai Negeri Sembilan Malaysia; b School of Management, Department of Management Studies, Vel Tech Rangarajan Dr Sagunthala RandD Institute of Science and Technology, University in Morai Tamil Nadu 600062 India asokan.vasudevan@newinti.edu.my; c College of Dentistry, University of Al Maarif Al Anbar 31001 Iraq; d Faculty of Technical Education, Hourani Center for Applied Scientific Research, Al-Ahliyya Amman University Amman Jordan; e Faculty of Engineering, Gokul Global University Sidhpur Gujarat India; f Department of CSE, School of Computing, Veltech Rangarajan Dr.Sagunthala R&D Institute of Science and Technology Avadi Chennai India; g Department of Chemistry, University Institute of Sciences, Chandigarh University Mohali Punjab India; h Head of the Department «Physics and Chemistry», Tashkent Institute of Irrigation and Agricultural Mechanization Engineers National Research University Tashkent Uzbekistan; i Scientific researcher of the University of Tashkent for Applied Science Tashkent Uzbekistan; j School of Engineering, Central Asian University Tashkent Uzbekistan; k Western Caspian University, Scientific researcher Baku Azerbaijan; l Research Follower, INTI International University 71800 Negeri Sembilan Malaysia; m Young Researchers and Elite Club, Islamic Azad University Tehran Branch Tehran Iran

## Abstract

Gold/graphene quantum dot (Au/GQD) hybrids combine chemically accessible carbon surfaces with metallic, plasmonic, and emissive gold components. This review examines their interfacial signal-transduction principles and the available evidence for metal-ion sensing. Material descriptors, including surface functionality, morphology, electronic alignment, and spatial separation, are related to optical attenuation, fluorescence modulation, and electrochemical response. Examples from characterization, photocatalysis, and non-metal assays illustrate selected structural or transduction processes; they are distinguished from direct evidence of metal-ion recognition. The metal-ion studies are evaluated according to component roles, measured responses, analytical performance, and mechanistic support. Particular attention is given to plasmon-modulated Cr^3+^ fluorescence, Cu^2+^/Cd^2+^ ratiometric detection, and reaction-generated reporting of Au^3+^. Their comparison reveals heterogeneous recognition mechanisms and incomplete analytical benchmarking rather than a universally validated recognition pathway. A general hybrid advantage remains unestablished without matched component and alternative-material controls. Adjacent Au/GQD assays provide design hypotheses whose transfer requires target-specific binding and interference validation. Persistent limitations include unresolved signal origins, concentration-dependent matrix effects, inconsistent detection-limit reporting, and poorly standardized interface characterization. The resulting synthesis provides a structured basis for interpreting Au/GQD responses and identifying evidence needed to connect interfacial changes with reliable metal-ion detection.

## Abstract 2

This review links Au/GQD interfacial architecture to metal-ion recognition and signal transduction. It identifies mechanistic evidence gaps and the need for matched controls to establish hybrid advantages.

## Graphical abstract

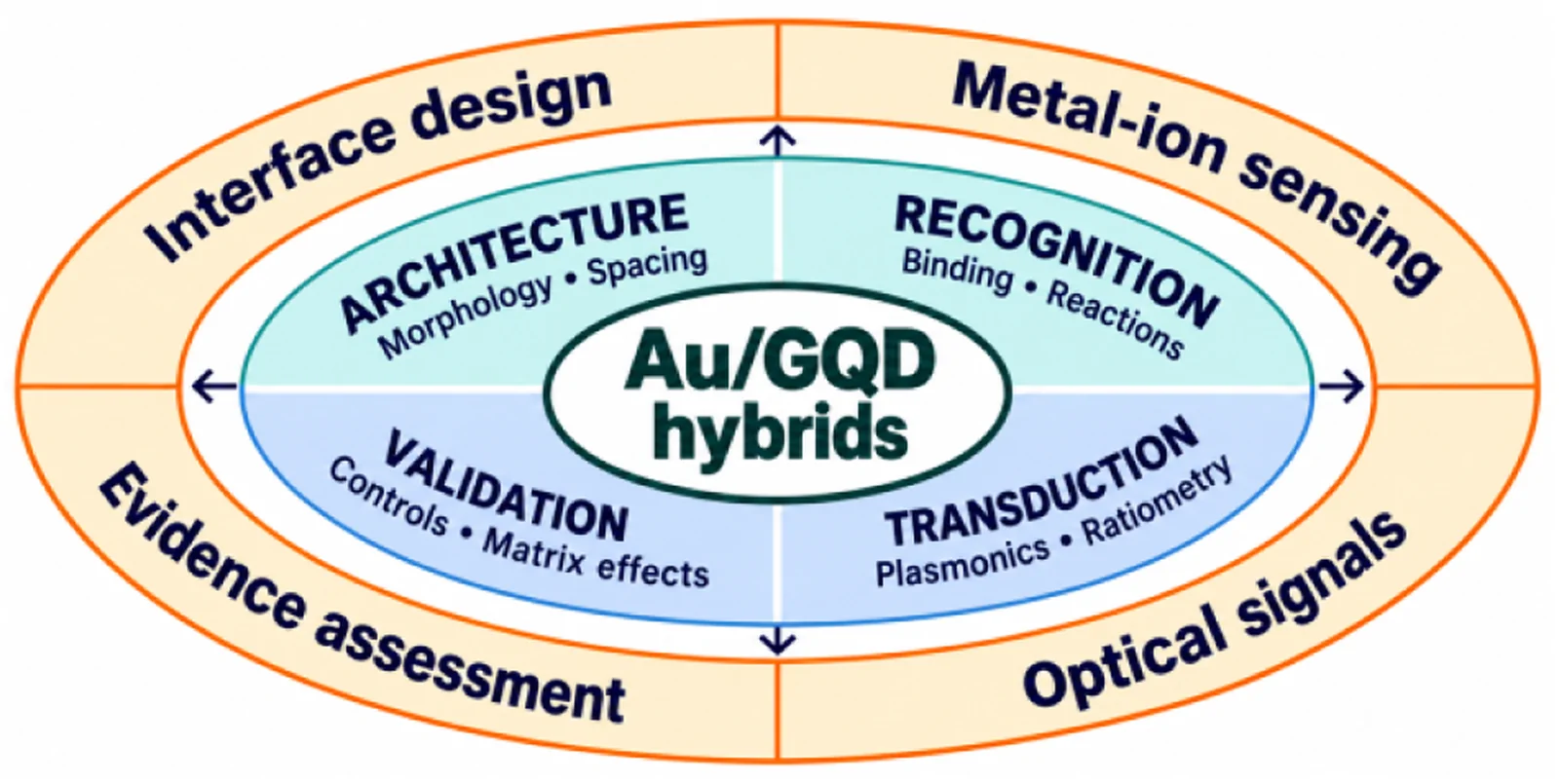

## 1 Introduction

Metal-ion ion contamination remains an analytically demanding problem because toxicity, persistence, chemical speciation, and matrix complexity place simultaneous requirements on sensitivity, selectivity, response speed, and operational simplicity. Conventional instrumental methods can provide high analytical accuracy, yet their dependence on centralized equipment, sample preparation, and trained personnel limits their suitability for decentralized or rapid monitoring.^1–5^ This has sustained interest in nanoscale sensing interfaces that can translate selective ion–surface interactions into optical or electrochemical signals.^6,7^ Within this context, graphene quantum dots (GQDs) and gold nanostructures represent a particularly compatible pair. GQDs offer size-dependent electronic structure, chemically accessible edge states, tunable surface functionality, and photoluminescent behavior; whereas gold provides high electrical conductivity, plasmonic activity, chemically addressable surfaces, and efficient coupling to interfacial charge-transfer processes.^8–10^ Their hybridization therefore creates a sensing interface in which recognition chemistry and signal generation can be structurally coupled rather than treated as independent functions.^11,12^ Au/GQD hybridization provides an opportunity to combine distinct recognition and reporting functions, but the coexistence of these components does not itself establish synergy. Depending on architecture, analyte interaction may alter fluorescence, charge-transfer kinetics, plasmonic response, or interfacial organization.^13–16^ Whether these changes provide an analytical advantage over the individual components or alternative hybrids must be established through matched comparisons for the same analyte. This versatility is also a source of ambiguity. Similar macroscopic signals can arise from different microscopic processes, including coordination, electrostatic adsorption, aggregation, surface-state redistribution, dielectric changes, or redox conversion. Consequently, high sensitivity alone does not establish a well-defined sensing mechanism. A central challenge in the field is therefore to determine how material structure, interfacial chemistry, and transduction pathway collectively govern analytical performance.^17–21^

The literature has largely approached Au/GQD sensing through individual platform development, emphasizing detection limits, linear ranges, selectivity tests, or proof-of-concept applications. Although these studies establish the functional diversity of Au/GQD architectures, they provide only a fragmented basis for design-level understanding. Three gaps are particularly important.^22–26^ First, the physicochemical properties of Au and GQDs are often discussed separately from the recognition-to-signal sequence that ultimately determines sensing behavior. Second, reported systems are frequently compared through isolated analytical figures of merit, despite substantial differences in interface architecture, transduction mode, and analyte-induced perturbation. Third, the distinction between direct metal-ion sensing evidence and transferable transduction concepts from adjacent Au/GQD platforms is rarely made explicit. These limitations hinder the development of generalized structure–property–performance relationships and make it difficult to identify which architectural features are genuinely transferable across sensing formats.

Previous reviews provide complementary foundations for interpreting GQD-based sensing. These include N-GQD coordination chemistry and fluorescence-quenching pathways,^27^ electrochemical detection of Cd(ii), Pb(ii), and Hg(ii) using GQD-based materials,^28^ and broader graphene-based platforms for environmental analysis.^29–31^ The distinction developed here concerns the unit of analysis: the Au–GQD interface and the sequence through which metal-ion recognition produces an analytical signal. Accordingly, established coordination, photophysical, and electrochemical mechanisms are considered in relation to the functional roles of the two hybrid components, rather than presented as independent explanations of sensor performance.

The proposed framework connects four questions: where the ion is recognized, which interfacial variable is perturbed, how that perturbation changes the measured signal, and what experimental evidence supports the proposed connection. Section 2 identifies relevant material descriptors, including surface functionality, Au morphology, interfacial separation, and electronic alignment. Section 3 relates these descriptors to recognition, optical or electrochemical transduction, and competing architectural effects. Section 4 distinguishes direct metal-ion sensing evidence from adjacent Au/GQD applications used to illustrate transferable transduction principles. This organization enables similar signals to be examined for different physical origins and similar compositions to be compared for different functional role allocations. The contribution is therefore a structured interpretive framework for assessing recognition–interface–signal relationships and their evidential limits, rather than a claim that the underlying sensing mechanisms are newly discovered or universally validated.

## 2 Physicochemical basis of Au–GQD hybrid sensing systems

### 2.1 Electronic and surface characteristics of gold nanostructures

The functional role of gold in Au–GQD hybrids originates from the convergence of metallic conductivity, high surface polarizability, tunable localized surface plasmon resonance, and a chemically accessible interface. At the nanoscale, collective oscillation of conduction electrons generates an electromagnetic response that is strongly dependent on particle size, geometry, interparticle separation, dielectric environment, and surface chemistry. These variables determine not only the spectral position and intensity of plasmonic modes but also the spatial distribution of the near field and the extent to which electronic excitation can perturb an adjacent semiconductor. Consequently, gold should not be treated merely as an inert conductive support; its contribution is governed by a coupled optical–electronic surface state whose behavior can change substantially with nanoscale architecture.

Gold nanospheres and nanostars illustrate how geometry changes plasmon–GQD coupling. For isolated nanospheres substantially smaller than the excitation wavelength, the optical response is generally dominated by a dipolar plasmon mode. Their smooth curvature produces a comparatively simple, polarization-dependent near-field distribution, without the sharp-tip field concentration characteristic of nanostars. Coupling to nearby GQDs consequently depends on sphere diameter, spectral overlap, and metal–GQD separation. Nanospheres can enhance GQD excitation or radiative emission under suitable conditions, but absorption and non-radiative energy dissipation can also suppress fluorescence; spherical geometry therefore does not imply intrinsically weak or exclusively quenching behavior.

Nanostars introduce additional geometric variables, including branch length, tip sharpness, branch number, and core size. Coupling between core and branch plasmon modes produces tunable resonances, while sharp tips concentrate the electromagnetic near field into localized hot spots. GQDs positioned near these tips may therefore experience stronger excitation fields than GQDs located near the core or outside the hot-spot region. However, shifting a branch resonance toward the near-infrared does not automatically improve coupling to visible-emitting GQDs: overlap with the relevant excitation or emission transition remains essential. Au nanostar/GQD architectures illustrate both resonance-matched optical enhancement and energy-transfer-based fluorescence suppression, depending on their interfacial organization.^32,33^ The stronger local field must therefore be balanced against metal-induced non-radiative losses, ligand-controlled separation, and heterogeneous GQD placement. Geometry-dependent electromagnetic enhancement should also be distinguished from interfacial electron transfer, which requires suitable electronic alignment and contact. Meaningful nanostar–nanosphere comparisons should consequently control surface chemistry, GQD loading, separation, and excitation conditions rather than attribute differences in fluorescence intensity to shape alone.

The high density of electronic states near the Fermi level also enables Au nanostructures to participate efficiently in interfacial charge redistribution. Contact with a carbon-based semiconductor may establish an interfacial potential gradient determined by differences in work function, surface dipoles, ligand chemistry, and local charge density.^32–34^ The resulting equilibrium state controls the energetic accessibility of electron-transfer processes without implying that charge transfer will necessarily dominate under all conditions. Surface-bound ligands further modify this landscape by changing interfacial distance, dielectric screening, steric accessibility, and local electrostatic potential. Thus, nominally identical Au cores can exhibit substantially different physicochemical behavior when their surface terminations differ.

These advantages coexist with intrinsic constraints. Plasmonic coupling becomes difficult to control when particles aggregate, while dense organic capping layers can electrically and spatially decouple gold from neighboring GQDs. Conversely, overly short interfacial distances may increase non-radiative dissipation rather than provide beneficial coupling.^35–37^ The physicochemical contribution of Au is therefore governed by a balance among metallic continuity, surface accessibility, interfacial separation, and dielectric environment. Establishing this balance provides the first constituent-level foundation for interpreting Au–GQD hybrids and leads naturally to the distinct semiconductor and surface-state characteristics contributed by GQDs.

### 2.2 Quantum confinement, surface states, and chemical heterogeneity of GQDs

Material identity must be established before invoking graphene-specific electronic behavior. In this review, the designation GQD refers to nanoscale graphene-like domains for which lateral dimensions, sheet-like morphology, and single- or few-layer character are supported by complementary structural measurements. Transmission electron microscopy and atomic force microscopy should be interpreted together with evidence of graphitic ordering; an isolated lattice fringe, an sp^2^-carbon XPS component, or blue photoluminescence alone does not establish GQD identity. Apparent AFM height also includes contributions from surface groups, adsorbates, and substrate interactions. Conversely, a bottom-up synthesis from citric acid does not by itself establish or exclude a graphene-like structure. Such preparations require particular attention to purification because molecular fluorophores and oligomeric products can contribute substantially to the measured emission. Accordingly, source-reported GQD terminology is retained when discussing individual studies, but insufficiently resolved materials are identified as carbon dots reported as GQDs. Their analytical results can remain informative, while graphene-specific confinement, band alignment, and transport mechanisms should be treated as provisional.

GQDs occupy an intermediate regime between extended sp^2^ carbon materials and molecular fluorophores. Their electronic structure is influenced simultaneously by quantum confinement, finite-domain effects, edge configuration, structural defects, heteroatom incorporation, and oxygen-containing surface functionalities. As a result, their optical and electronic properties cannot be reduced to particle size alone. Variations in the relative contributions of π-conjugated domains and localized surface states alter the density and energetic distribution of accessible electronic levels, thereby affecting absorption, photoluminescence, carrier localization, and interfacial charge susceptibility. This multidimensional dependence is central to the use of GQDs in hybrid sensing systems because it couples structural heterogeneity directly to functional heterogeneity.

Surface chemistry is particularly important. Carboxyl, hydroxyl, carbonyl, amino, and other functional groups modify local charge density, hydrophilicity, acid–base behavior, coordination potential, and the degree of electronic localization at the GQD perimeter. These groups can therefore influence both the interaction of GQDs with metallic surfaces and their physicochemical response to changes in the surrounding medium.^38–40^ Heteroatom doping adds another level of control by perturbing the electronic density of states and introducing chemically non-equivalent binding environments. Nevertheless, such modifications rarely alter only one property. Increasing defect density, for example, may create additional chemically accessible sites while simultaneously increasing non-radiative relaxation pathways. Similarly, stronger surface functionalization can improve colloidal stability but disrupt the effective π-conjugated domain.

This coupling produces an important structure–property constraint: chemical accessibility, fluorescence efficiency, electronic transport, and surface-state density cannot generally be optimized independently. GQDs with high emissive intensity are not necessarily those with the highest interfacial electronic responsiveness, and materials with abundant coordination sites may exhibit broader energetic disorder. The relevant design variable is therefore not the maximization of a single GQD property but the controlled distribution of electronic and chemical states.^41–43^ This constituent-level complexity becomes more consequential when GQDs are placed in direct proximity to Au, where the properties of neither component remain fully independent.

Fig. 1 provides qualitative XPS evidence of changes in the surface chemical environment when N-GQDs are converted into graphene@Au.^42^ Panel (a) identifies C, N, O, and Au in the hybrid, but the displayed survey spectra do not provide sensitivity-factor-corrected atomic concentrations from which reliable C/O and C/N ratios can be calculated. Peak heights alone are insufficient for this comparison because elemental quantification requires background-subtracted integrated intensities and appropriate instrumental sensitivity factors.

**Fig. 1:**
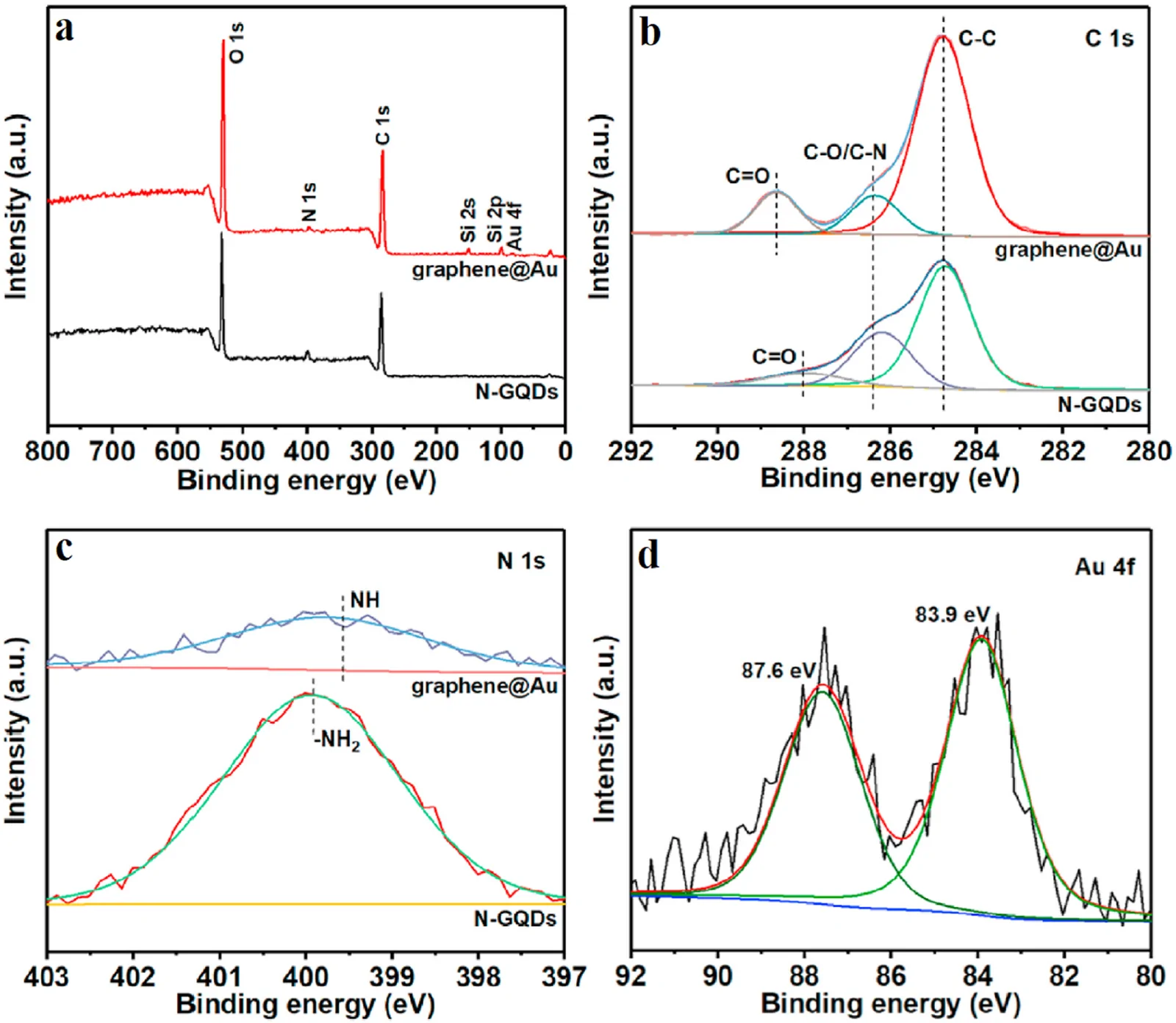
XPS characterization of N-GQDs and graphene@Au: (a) survey spectra, (b) C 1s, (c) N 1s, and (d) Au 4f regions. Reproduced with permission from ref. 42. © 2021 Elsevier B.V.

Panel (b) shows changes in the C 1s envelope, including contributions assigned to C–C, C–O/C–N, and carbonyl-containing environments. Panel (c) shows a change in the N 1s environment from the precursor amino-group assignment toward an NH-containing environment in graphene@Au. Together, these observations are consistent with the proposed condensation-related reorganization of surface functionalities, although the overlapping C–O/C–N contribution and the absence of numerical component areas limit quantitative attribution. Panel (d) shows the Au 4f doublet at approximately 83.9 and 87.6 eV, consistent with metallic Au. The figure therefore supports changes in bonding environments and Au incorporation, but does not independently quantify oxygen or nitrogen loss or establish the extent of chemical conversion. Moreover, changes in C/O or C/N, if available, would complement chemical-state analysis rather than uniquely prove amide formation.

### 2.3 Interfacial electronic equilibration and nanoscale coupling in Au–GQD hybrids

Hybridization of Au nanostructures with GQDs creates an interface whose properties cannot be described as a simple superposition of those of the isolated constituents. Upon contact, electronic equilibration is governed by the relative work functions, Fermi-level positions, surface dipoles, defect states, and chemical terminations of the two phases. Charge redistribution can consequently modify the local electrostatic potential and the occupation of interfacial electronic states. The magnitude and direction of this redistribution are sensitive to GQD composition and Au surface chemistry, meaning that the term “Au–GQD interface” describes a family of energetically distinct interfaces rather than a single standardized configuration.

A quantitative interpretation requires specifying both the energy reference and the material preparation. Taking the vacuum level as zero, an Au work function of approximately 5.1 eV corresponds to a Fermi-level energy of approximately −5.1 eV.^44^ GQDs do not possess a universal work function: values of approximately 3.65 eV and 4.48 eV have been reported for separately prepared GQD samples using Kelvin-probe force microscopy and ultraviolet photoelectron spectroscopy, respectively.^45^ Relative to the Au reference, these values imply nominal pre-contact work-function differences of approximately 1.45 and 0.62 eV. Under an idealized contact model, this ordering favors initial electron redistribution from GQDs toward Au; however, interface dipoles, ligand barriers, and electronic-state pinning can modify the actual alignment. These differences are therefore neither measured interfacial potential drops nor photoinduced electron-transfer driving forces. Furthermore, work function identifies the Fermi-level position rather than the valence- or conduction-band edge. Sample-specific occupied and unoccupied energy levels must be established independently before assigning a particular excited-state transfer pathway.

Spatial separation introduces competing electronic and optical length scales. Here, *d* denotes the effective separation between the Au surface and the relevant GQD electronic or emissive state, rather than the particle center-to-center distance. Across thin insulating spacers, direct electron tunnelling is principally relevant at subnanometre to approximately 1–2 nm separations and decreases approximately exponentially, *k*_ET_ ∝ exp(−*βd*), where *β* depends on the spacer chemistry and barrier height. Measurements on ligand-separated Au clusters demonstrate this exponential attenuation, although they do not establish a universal Au–GQD distance threshold.^46^ At intermediate separations, approximately 5–10 nm provides a useful design window for examining plasmon-mediated enhancement. Enhancement is nevertheless conditional on Au geometry, spectral overlap, emitter orientation, and the balance between radiative and non-radiative decay; it is not guaranteed throughout this interval.

Non-radiative energy transfer to Au can persist beyond the range of appreciable direct tunnelling. Nanometal surface energy transfer has been demonstrated over approximately 22 nm in molecular-donor/Au systems, with an approximately inverse-fourth-power distance dependence under the conditions supporting that model.^44–46^ Consequently, separations of tens of nanometres cannot automatically be regarded as an uncoupled regime. These overlapping processes explain why decreasing separation can strengthen excitation or charge exchange while simultaneously increasing dissipative decay. A non-monotonic fluorescence response can therefore emerge from their competition, but its maximum must be established for the specific architecture rather than inferred from nominal distance alone. The cited length scales provide mechanistic benchmarks from related systems, not experimentally verified boundaries for every Au–GQD hybrid.

The surrounding medium can modify both the effective Au–GQD separation and the coupling processes operating at that separation. Changes in pH alter the protonation of GQD edge groups and ionizable Au-bound ligands. For carboxyl-rich surfaces, increasing pH above the relevant acid-dissociation range increases the population of negatively charged carboxylate groups. Consistent with this chemical sensitivity, pH-dependent deprotonation of oxygen-containing GQD groups has been discussed during GQD–Au hybrid synthesis.^48^ In a dispersed sensing system, greater negative charge on both components can strengthen electrostatic repulsion and reduce close approach, whereas negatively charged GQDs may associate more strongly with positively charged Au-bound ligands. Acidification can reduce carboxylate-mediated repulsion, but protonation of amine-containing ligands may simultaneously strengthen attraction. The direction of the distance change therefore depends on the surface chemistry of both components.

Ionic strength modifies these interactions through electrostatic screening. Under the dilute-electrolyte approximation, the Debye screening length decreases approximately as the inverse square root of ionic strength. Increasing salt concentration can consequently weaken repulsion between similarly charged components, permitting closer approach or aggregation when steric stabilization is insufficient. For oppositely charged components, however, screening can weaken electrostatic association and reduce hybrid assembly. These effects primarily alter the distribution of accessible separations; they do not establish a universal change in the intrinsic distance dependence of electromagnetic coupling. In covalently linked or rigidly spaced hybrids, the physical gap may remain approximately fixed even when ionic strength changes surface potential or interfacial screening.

These conditional effects explain how solution conditions can modify an apparent non-monotonic optical response. If screening or protonation moves GQDs toward a favorable near-field region, excitation enhancement may increase; further approach can instead favor metal-induced non-radiative losses. Meanwhile, pH can independently modify GQD emissive states, and aggregation can shift Au plasmon bands and spectral overlap.^44,45^ Fluorescence changes therefore cannot, by themselves, demonstrate movement toward or away from an optimal separation. Measurements of surface charge, colloidal size, absorption, and fluorescence lifetime under matched conditions are needed to distinguish distance redistribution from altered surface photophysics. This interpretation provides a conditional mechanistic explanation rather than a quantitatively established pH–distance or salt–distance relationship for all Au–GQD hybrids.

Fig. 2 summarizes structural and spectroscopic observations reported for a GQD–Au hybrid.^48^ Panel (a) shows nanoparticles and an HR-TEM inset containing periodic features labelled in the source as Au(111), with a spacing of approximately 0.233 nm, and graphite (100), with a spacing of approximately 0.242 nm. The former is consistent with the expected Au(111) spacing. However, the latter should not be treated as an unambiguous graphite (100) assignment: the conventional graphite (100) interplanar spacing is approximately 0.213 nm, whereas the in-plane lattice parameter is approximately 0.246 nm. Accordingly, the 0.242 nm annotation is retained as a source-reported measurement whose crystallographic indexing remains uncertain. Verification would require calibrated image analysis and supporting diffraction or Fourier-transform evidence; the image alone does not justify assigning the discrepancy to strain or defects. Panel (b) shows an additional visible absorption band in the hybrid relative to the GQDs, consistent with a contribution from Au nanoparticle plasmonic absorption. Together, these observations support the reported Au-containing hybrid architecture, but do not independently establish interfacial bonding or the direction of charge redistribution.

**Fig. 2:**
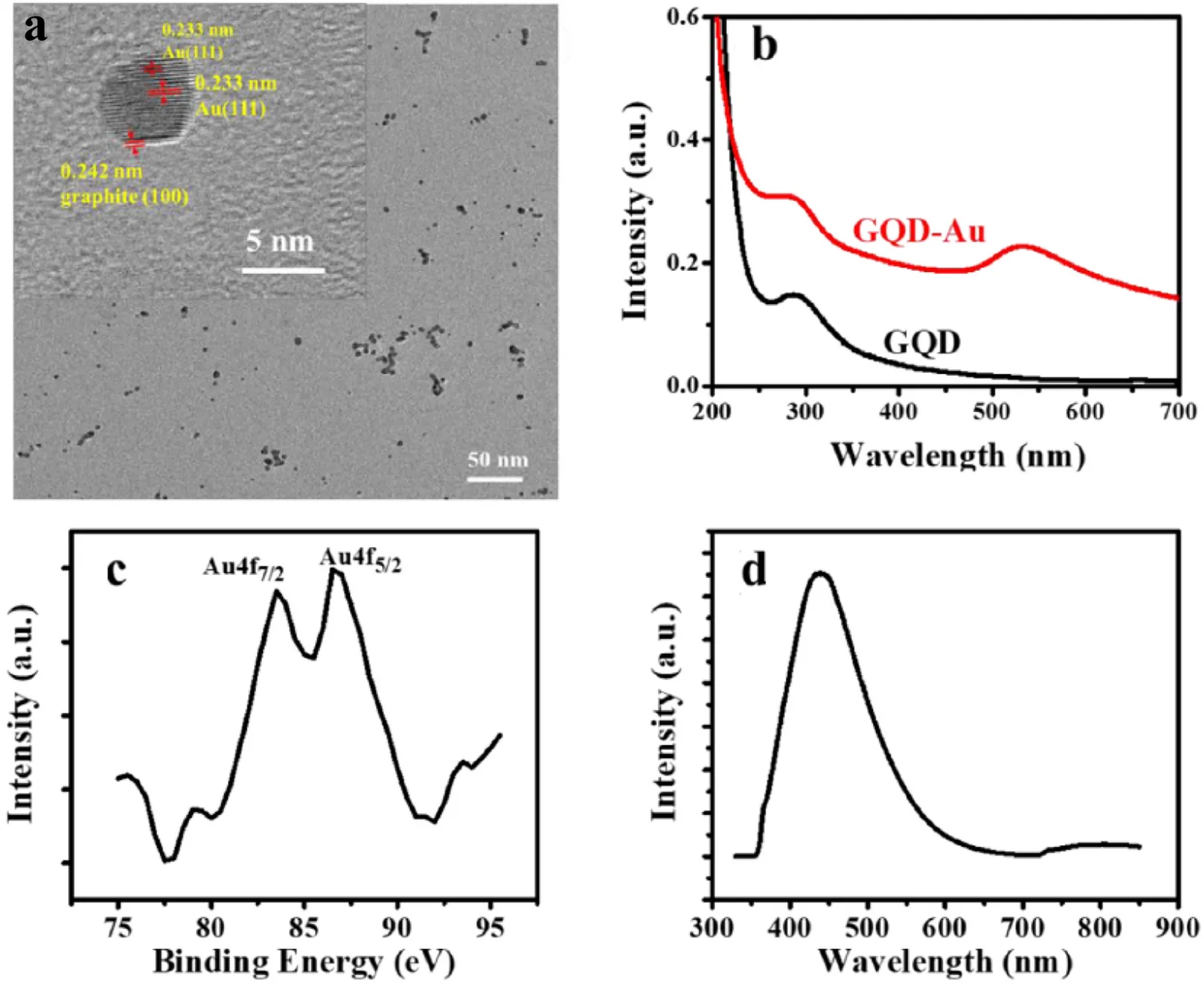
Characterization of GQD–Au hybrids: (a) HR-TEM, (b) UV-vis spectra, (c) Au 4f XPS, and (d) PL under 270 nm excitation. Source annotations are retained; the graphite (100) indexing and definitive Au chemical-state assignment remain unverified. Reproduced with permission from ref. 48. © 2020 Elsevier B.V.

Panel (c) displays the Au 4f region, with features previously quoted at approximately 83.5 and 86.8 eV. Their apparent separation of approximately 3.3 eV differs from the conventional Au 4f spin–orbit splitting of approximately 3.67 eV. Given the substantial spectral fluctuations and the absence of displayed constrained fit or peak-position uncertainties, these values are not sufficiently established to confirm a metallic Au chemical state or quantify an interfacial binding-energy shift. Reliable verification would require the original spectrum, a documented energy reference, and physically justified doublet fitting, including an approximately 3.67 eV separation and an appropriate 4f_7/2_ : 4f_5/2_ integrated-area ratio, conventionally 4 : 3. These fitting criteria are requirements for verification, rather than evidence that such verification has been performed here. Panel (d) shows luminescence under 270 nm excitation, but steady-state emission alone cannot distinguish charge transfer, plasmonic modulation, surface-state changes, and non-radiative losses. Fig. 2 is therefore used as a qualitative characterization example, with explicit limitations on chemical-state assignment, crystallographic indexing, and mechanistic interpretation.

### 2.4 Intrinsic physicochemical constraints and conceptual gaps in Au–GQD hybrid systems

A central limitation of Au–GQD hybrid systems is that several physicochemical variables influencing their observable response are strongly coupled and experimentally difficult to isolate. GQD size distribution, edge structure, surface oxidation, heteroatom content, Au morphology, ligand coverage, interfacial distance, and aggregation state can change simultaneously during preparation or environmental exposure. The resulting response therefore reflects an ensemble of interdependent properties rather than a single controllable parameter. This complicates the establishment of universal structure–property relationships and makes nominal material composition an insufficient descriptor of hybrid behavior.

Another conceptual difficulty arises from the multiplicity of competing relaxation and charge-redistribution pathways. Changes in emission intensity or interfacial conductivity may originate from alterations in carrier transfer, surface trapping, dielectric screening, plasmonic coupling, aggregation, or chemical modification of surface states.^49–51^ These processes can operate concurrently and may produce similar macroscopic observables. A rigorous physicochemical description therefore requires distinguishing correlation from mechanistic causality. Assigning a response to one interaction solely from the direction of signal change risks oversimplifying a system in which several pathways are energetically plausible.

Reproducibility is further constrained by the poorly standardized nature of GQDs. Materials classified under the same designation can differ substantially in carbon-core size, oxidation degree, defect density, photoluminescence origin, and surface functionality. Similar ambiguity exists for Au components when differences in nanosphere diameter, cluster state, film morphology, or surface ligands are not explicitly incorporated into interpretation.^52–54^ These variations limit the transferability of mechanistic conclusions across nominally similar hybrid systems.

The principal conceptual gap is therefore not the absence of possible interaction mechanisms but the lack of a sufficiently unified set of physicochemical descriptors linking composition and nanoscale structure to interfacial state and ultimately to functional response. A robust foundation requires distinguishing directly contacted interfaces, ligand-separated assemblies, coated particles, supported films, and multicomponent mixtures. Interfacial electronic coupling should be invoked only where the architecture and characterization support it; complementary signal generation in a mixture should be evaluated separately. Likewise, graphene-specific explanations require evidence that the carbon component retains a graphene-like structure after synthesis and assembly.

### 2.5 Synthesis routes and architectural types of Au–GQD systems

Synthesis and assembly determine whether Au and the carbon component form a directly contacted interface, a ligand-separated conjugate, a coated particle, an electrode-supported composite, or a multicomponent dispersion. These architectures differ in interfacial separation, contact area, accessible recognition sites, and electronic connectivity. Consequently, the preparation route is a mechanistically relevant descriptor rather than background information, and nominal Au/GQD composition alone cannot establish the operative coupling pathway.

*In situ* reduction uses preformed carbon dots as reducing and stabilizing components during conversion of an Au precursor into metallic nanoparticles. A representative preparation reduces HAuCl_4_ using preformed, citric-acid-derived dots at room temperature.^48^ This route can place newly formed Au close to the carbon surface, but nucleation conditions, precursor ratio, pH, and reaction time may alter Au size, coverage, and aggregation. Carbon-surface oxidation or reorganization during reduction must also be considered. Direct electronic contact should therefore be established by characterization rather than inferred solely from simultaneous formation or reagent composition.

Post-synthetic assembly offers a different interface. Thiol-containing linkers can anchor to Au, while terminal amine groups provide sites for association or further coupling with functionalized GQDs; cysteamine-functionalized Au/GQD conjugates illustrate this approach.^60^ Where carbodiimide-mediated coupling is used, amide formation can connect carboxyl-bearing dots to amine-bearing linkers. However, Au–S anchoring, amide formation, amine adsorption, and electrostatic association represent distinct chemical interactions and require different evidence. Linker length and coverage introduce a separation barrier that can preserve fluorescence while attenuating direct electron transfer. Changes in protonation or ligand displacement may additionally alter assembly stability.

Wrapped and core–shell structures must be distinguished from particles bearing isolated surface-bound dots. Microplasma synthesis has produced Au-core/carbon-shell nanohybrids described as GQD-wrapped Au nanoparticles,^36^ whereas another preparation converts N-GQDs into graphene on Au.^42^ Such transformations can change the identity and connectivity of the carbon phase. Shell continuity, thickness, composition, and permeability determine whether analytes encounter the carbon coating, exposed Au, or both. A core–shell designation therefore requires spatially resolved structural evidence; elemental coexistence alone is insufficient.

Films and electrode-supported composites introduce collective organization beyond individual particle interfaces. AuNP/GQD/graphene-oxide electrode films and Au/GQD sensing films exemplify these architectures.^35^ Their responses can depend on film thickness, porosity, Au distribution, substrate contact, and transport through the deposited layer. Consequently, lower impedance or enhanced optical response cannot automatically be attributed to a single Au–GQD junction without separating changes in loading, accessible area, and film connectivity.

Finally, AuNC–GQD dispersions may operate through complementary emission channels without requiring a permanent, strongly coupled interface. The Cu^2+/^Cd^2+^ probe in Section 4.1 was reported as an electrostatically associated nanomixture. Its mixing ratio and association stability are therefore relevant descriptors, and a ratiometric response alone does not establish interfacial charge transfer. Ultrasmall emissive AuNCs must also be distinguished from plasmonic Au nanoparticles; localized surface plasmon enhancement should not be assigned to the AuNC channel without supporting optical evidence. These architectural distinctions define the conditions under which the subsequent interfacial framework can be applied.

Table 1 reinforces the conceptual logic by separating intrinsic material descriptors from their physicochemical consequences and design constraints, rather than treating Au and GQDs as additive components. In particular, it highlights that hybrid behavior emerges from coupled parameters such as energy alignment, interfacial distance, state density, and environmental screening, which cannot be optimized independently. This framework provides a clear foundation for subsequent analysis of recognition and transduction processes without prematurely entering application-specific evidence.

**Table 1:** Conceptual physicochemical descriptors governing Au–GQD hybrid sensing interfaces

Physicochemical factor	Primary structural origin	Fundamental role	Functional consequence	Intrinsic limitation	Design significance	Ref.
Au morphology	Size, geometry, curvature	Governs plasmonic and electronic response	Alters near-field distribution and surface accessibility	Morphology-dependent variability	Requires controlled nanoscale geometry	30 and 36
Au surface chemistry	Ligands and surface termination	Modifies local potential and interfacial accessibility	Tunes electronic communication with GQDs	Dense capping can decouple interfaces	Ligand density should balance stability and coupling	31 and 39
GQD size and confinement	Finite sp^2^ domains	Controls electronic-state distribution	Influences absorption, emission, and carrier localization	Size effects are coupled with edge chemistry	Size alone is an insufficient descriptor	29 and 47
GQD edge functionality	Carboxyl, hydroxyl, carbonyl, amino groups	Regulates local charge and coordination potential	Changes hydrophilicity and interfacial responsiveness	Excess functionalization can disrupt π-conjugation	Functional-group density must remain electronically compatible	34 and 50
GQD defect density	Vacancies and structural disorder	Generates localized electronic states	Modifies surface-state population and carrier trapping	Defects may increase non-radiative relaxation	Controlled, not maximal, defect density is preferable	28 and 47
Heteroatom incorporation	N, S, or other dopants	Perturbs electronic density and surface polarity	Creates chemically non-equivalent local environments	Doping can broaden energetic heterogeneity	Dopant identity and distribution require control	31 and 44
Au–GQD energy alignment	Work-function and Fermi-level differences	Drives interfacial electronic equilibration	Determines charge redistribution and local potential	Strongly dependent on surface state	Energy-level compatibility is a key interface descriptor	40 and 52
Interfacial separation	Physical distance and spacer layer	Controls electronic and electromagnetic coupling	Regulates coupling strength between both phases	Too short promotes dissipation; too long weakens coupling	An intermediate coupling window is required	37 and 44
Interfacial state density	Defects, terminations, contact regions	Governs occupation of localized states	Modifies electronic communication across the interface	Highly heterogeneous and difficult to quantify	Should be characterized alongside composition	32 and 39
Environmental screening	pH, ionic strength, dielectric environment	Alters charge screening and protonation	Shifts local electrostatic and energetic conditions	Interface state becomes condition-dependent	Operating environment must be treated as part of the material system	45 and 48
Aggregation and structural heterogeneity	Particle clustering and batch variability	Changes effective contact and optical/electronic coupling	Produces non-uniform functional response	Limits reproducibility and mechanistic transferability	Structural uniformity is essential for reliable interpretation	50 and 53
GQD thickness and layer number	Out-of-plane thickness, layer number, and stacking	Modifies interlayer interactions and accessible surface geometry	Can alter electronic states, emission, and contact with Au	Apparent AFM height includes surface groups and adsorbates; thickness effects overlap with chemistry	Report thickness separately from lateral size and distinguish individual-dot thickness from coating thickness	48 and 53

GQD thickness and synthesis history provide additional descriptors that cannot be inferred from lateral size or nominal composition. Individual-dot thickness and layer number should be distinguished from the thickness of a GQD coating deposited on Au, because only the latter directly describes the spatial extent of that coating. Apparent atomic force microscopy height also includes contributions from surface functionalization and adsorbates and should not be converted into layer number without qualification. Similarly, synthesis and hybrid assembly affect multiple coupled variables: top-down cutting or bottom-up formation influences carbon structure and surface termination, while *in situ* Au growth or assembly of preformed components influences contact geometry and residual ligands. These descriptors therefore support interpretation of measured interface properties rather than a universal ranking of thicker *versus* thinner GQDs or one synthesis route over another.

## 3 Interfacial recognition and signal-transduction frameworks in Au–GQD hybrids

### 3.1 Recognition-site architecture and interfacial selectivity logic

The analytical behavior of an Au–GQD sensing interface is determined first by how target ions perturb chemically distinguishable recognition sites rather than by the optical or electrical readout itself. From a design perspective, recognition should therefore be treated as an interfacial free-energy problem involving coordination affinity, electrostatic attraction, competitive solvation, ligand accessibility, surface protonation, and steric confinement. The relevant question is not simply whether a metal ion can bind to the hybrid surface, but whether its binding event produces a sufficiently differentiated interfacial perturbation relative to chemically similar competing ions. Selectivity consequently emerges from the coupling between binding thermodynamics and the spatial organization of active sites.

The Au and GQD domains provide chemically non-equivalent environments. Gold surfaces can support ligand-mediated coordination and strong interactions with selected donor groups, whereas GQD edges and defect-rich regions contain oxygen-, nitrogen-, or sulfur-containing functionalities with distinct Lewis basicity and protonation behavior.^55–57^ Their coexistence can create a distributed recognition landscape in which the analyte interacts preferentially with one domain, bridges both domains, or reorganizes the local interfacial charge distribution.

On the GQD side, candidate coordination sites include accessible carboxylate oxygen atoms and appropriately configured nitrogen or sulfur donor groups. Their availability depends on protonation, local bonding, and competition from solvent or dissolved ligands. Total oxygen or nitrogen content is therefore not equivalent to a measured population of active binding sites, and heteroatom incorporation alone does not establish selective coordination. Importantly, GQDs may instead function as an emissive reference, support, or reducing component. These roles must be distinguished from direct ion recognition using component controls and chemical-state evidence.

However, increasing the density of binding functionalities does not necessarily improve discrimination. Excessive surface functionalization can broaden the distribution of binding energies, increase nonspecific adsorption, and reduce access to electronically active regions. Recognition efficiency must therefore be evaluated together with chemical heterogeneity.

A useful generalized design principle is that selectivity should be engineered through energetic differentiation rather than maximum binding strength. A thermodynamic example clarifies this distinction. Consider an idealized, homogeneous population of interfacial sites, L, at which a target ion, M, and an interfering ion, X, compete through reversible 1 : 1 binding. Their relative affinity is described by 
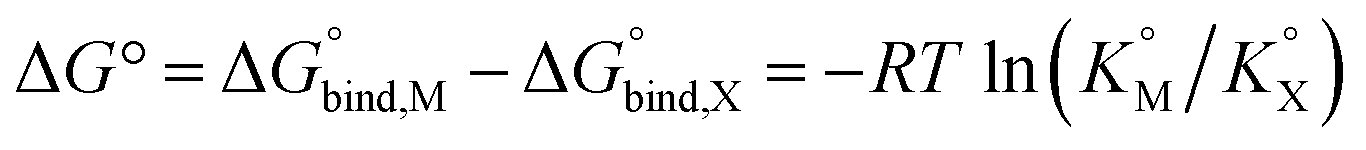
, where 
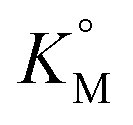
 and 
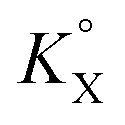
 are dimensionless binding equilibrium constants defined using consistent standard states. As an illustrative calculation rather than a measured result for a particular Au–GQD sensor, a 100-fold affinity preference for M corresponds to Δ*G*° ≈ −11.4 kJ mol^−1^ at 298 K. Under the competitive-site model, 
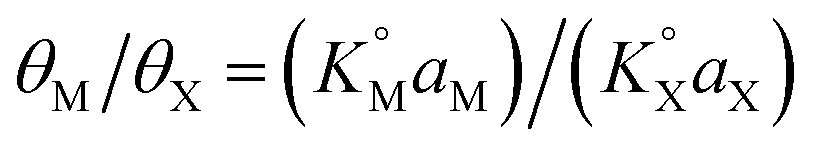
, where *θ* denotes site occupancy and a denotes free-ion activity.

Thus, equal free-ion activities yield a 100-fold target-to-interferent occupancy ratio, but a 100-fold excess of the interferent reduces that ratio to unity. Engineering energetic differentiation therefore means arranging donor groups, coordination geometry, and the local electrostatic environment to preferentially stabilize target binding relative to competing binding. Increasing both affinities by the same factor leaves their ratio unchanged. In practical Au–GQD interfaces, protonation, solvation, ion speciation, and site heterogeneity can modify this preference; moreover, preferential occupancy produces analytical selectivity only when target binding is effectively coupled to the measured signal.

Very strong but nonspecific coordination may suppress reversibility and amplify matrix effects, whereas moderately strong, geometrically and electronically matched binding can generate a more interpretable response. Recognition layers should also preserve sufficiently direct communication with the Au–GQD interface; otherwise, chemically selective binding may become electronically silent.^58–60^ Accordingly, the first mechanistic requirement is the conversion of molecular-scale ion recognition into a reproducible perturbation of interfacial energy, charge density, or spatial configuration. Once this recognition event is established, the next design question concerns how that perturbation is translated into an optical or electrochemical signal.

### 3.2 Signal-transduction pathways: fluorescence modulation and electrochemical conversion

Signal generation in Au–GQD hybrids can be rationalized as a sequence in which analyte recognition modifies one or more interfacial variables that subsequently alter an observable optical or electrical response. For fluorescence-based transduction, the central variables include excited-state population, radiative and non-radiative decay rates, energy transfer, electron transfer, surface-state occupancy, and the local electromagnetic field. The analytical response therefore depends on the balance between competing pathways rather than on fluorescence enhancement or quenching as isolated phenomena. A target-induced decrease in emission, for example, may arise from increased non-radiative relaxation, interfacial electron transfer, aggregation-induced coupling, or altered surface trapping, and these mechanisms are not functionally equivalent from a design standpoint.

Electrochemical transduction follows a related but distinct logic. Here, ion recognition must alter charge-transfer kinetics, interfacial capacitance, surface potential, redox accessibility, or mass-transfer behavior sufficiently to produce a measurable electrical change.^61–63^ The Au component can provide conductive pathways and electronically accessible surface states, whereas GQDs can regulate interfacial charge density and molecular accessibility. The design challenge is to ensure that the recognition event perturbs the rate-limiting electrochemical step rather than a secondary process with weak analytical leverage. Consequently, a highly conductive hybrid is not automatically an effective sensor if target binding produces little modulation of the interfacial electron-transfer landscape.

Metal-ion electroanalysis also includes accumulation–stripping pathways that differ fundamentally from conductivity enhancement or recognition-layer blocking. In anodic stripping voltammetry, a controlled deposition potential first drives reduction and accumulation of electroactive metal species, followed by an anodic scan that oxidizes the deposit and generates the analytical signal. Deposition potential and duration, transport conditions, electrolyte composition, and accessible Au area therefore influence accumulation efficiency, co-deposition, and interference. Underpotential deposition describes deposition at potentials more positive than those required for bulk deposition under comparable conditions, reflecting favorable interactions between the deposited species and the substrate; it should not be assumed for every metal–Au combination.^88^

For mercury, reduction of Hg(ii) and subsequent Au–Hg amalgamation provide an established route for accumulation or modification of gold's optical response.^88,89^ This pathway is distinct from ligand-mediated recognition and does not constitute a general explanation for all metal ions. In Au/GQD electrodes, a GQD layer could alter ion partitioning, transport, or access to deposition sites, but these contributions require deposition–stripping controls. Improved conductivity or hydrazine oxidation alone cannot demonstrate improved metal preconcentration or stripping performance. Accordingly, these established gold-based routes provide a mechanistic benchmark rather than evidence that the Au/GQD systems discussed here have experimentally realized them.

Optical and electrochemical pathways should therefore be distinguished by the variable they interrogate. Measured fluorescence reflects both the behavior of the emissive population and optical attenuation along the excitation and emission paths, whereas electrochemical measurements probe charge transport, interfacial kinetics, and mass-transfer processes. Their coexistence within the same material platform is valuable only when the relevant perturbations are mechanistically separable.^64–67^ The general design principle is thus to maximize the coupling efficiency between recognition and the chosen transduction variable, rather than maximizing intrinsic fluorescence intensity or conductivity alone. This requirement leads to the broader issue of how nanoscale architecture controls the transfer of information from binding site to measurable signal.

Fig. 3 illustrates fluorescence attenuation in a GQD–AuNP system used in a gentamicin assay.^65^ The displayed titration varies AuNP concentration rather than gentamicin concentration; therefore, these panels characterize absorber-dependent attenuation rather than directly demonstrating analyte-triggered transduction or metal-ion recognition. Panel (a) shows overlap between GQD emission and AuNP absorption, establishing a spectral basis for secondary inner filter attenuation of emitted light. Assessing primary inner filter attenuation additionally requires determining AuNP absorption at the excitation wavelength. Panel (b) shows nearly unchanged fluorescence lifetimes of 9.18 ± 0.03 ns without AuNPs and 9.16 ± 0.19 ns with AuNPs. This observation argues against a substantial dynamic-quenching contribution to the observed emissive population under the measured conditions, because dynamic quenching introduces an additional excited-state deactivation pathway and should shorten its lifetime. For a homogeneous fluorophore population undergoing purely dynamic quenching, the absorption-corrected intensity and lifetime ratios follow *F*_0,corr_/*F*_corr_ = *τ*_0_/*τ* = 1 + *k*_q_*τ*_0_[Q], where *k*_q_ is the bimolecular quenching rate constant and [Q] is quencher concentration.

**Fig. 3:**
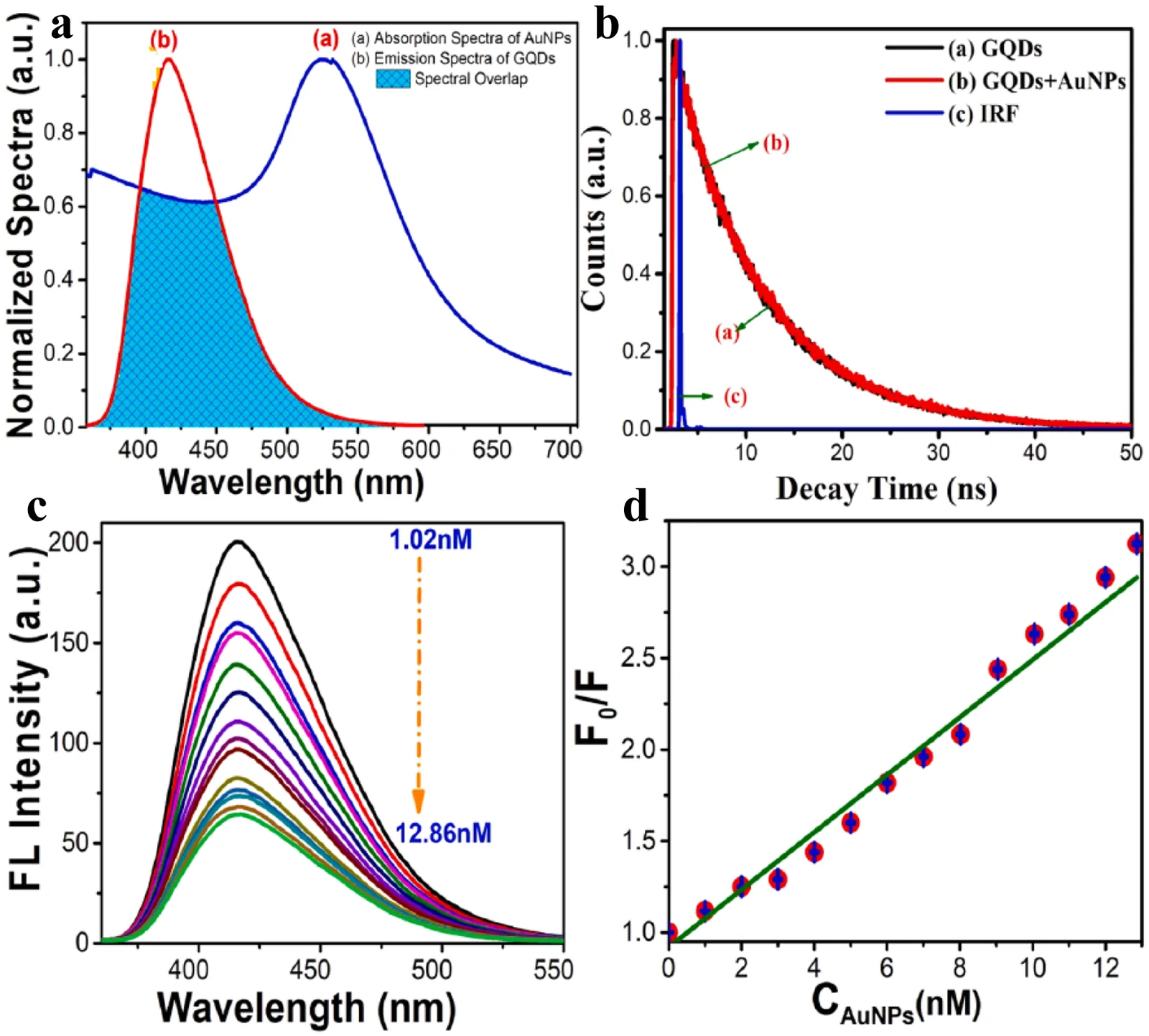
Optical attenuation in a GQD–AuNP system used for gentamicin sensing: (a) spectral overlap, (b) fluorescence decays, (c) AuNP-concentration-dependent emission, and (d) Stern–Volmer-type plot. Reproduced with permission from ref. 65. © 2022 Elsevier B.V.

Nevertheless, the lifetime comparison does not establish that dynamic quenching is entirely absent. A small contribution could remain unresolved within the measurement uncertainty, while heterogeneous GQD populations could exhibit changes not adequately represented by a single reported lifetime. Transient encounters with AuNP surfaces could potentially introduce non-radiative deactivation, but the available data do not quantify this contribution. Importantly, unchanged lifetime is also compatible with static quenching: non-emissive ground-state associations can reduce fluorescence intensity while leaving the lifetime of the remaining emissive population unchanged. Consequently, the lifetime measurements cannot independently distinguish IFE from static quenching or establish their relative contributions.

Panel (c) shows approximately 68% fluorescence attenuation as AuNP concentration increases from 1.02 to 12.86 nM, and panel (d) presents an approximately linear intensity-based Stern–Volmer-type relationship. This linearity is descriptive rather than mechanistically diagnostic; it does not establish IFE, collisional quenching, or the absence of static association. A more discriminating assessment requires concentration-matched absorption spectra and fluorescence-decay measurements, together with intensity corrections appropriate to the excitation and emission wavelengths and measurement geometry. Comparing mixture absorption with the concentration-weighted component spectra can help identify association or aggregation, although spectral changes alone do not prove static quenching. Excitation-wavelength checks should account for intrinsic GQD excitation dependence, while optical-path-length or front-face measurements should preserve sample composition. Establishing analyte-mediated transduction further requires matched measurements with and without gentamicin at fixed AuNP and GQD loading. Accordingly, Fig. 3 provides evidence compatible with an IFE contribution, while its dominance and possible concurrent static or dynamic contributions remain unresolved by the displayed measurements.

### 3.3 Structure–function coupling and architectural control of analytical response

The relationship between nanoscale architecture and sensing behavior can be formulated as a signal-propagation problem across three hierarchical levels: recognition-site accessibility, interfacial coupling, and macroscopic readout. Structural parameters such as Au–GQD separation, particle distribution, surface coverage, GQD loading, ligand thickness, aggregation state, and conductive connectivity determine how effectively an analyte-induced perturbation is transmitted across these levels. The relevant structure–function relationship is therefore not monotonic. Architectures that maximize one favorable property can simultaneously suppress another.

Short Au–GQD separation may strengthen electronic or electromagnetic coupling but can also increase non-radiative energy dissipation. High Au loading can improve electrical connectivity yet reduce the fraction of optically active GQD surface or introduce plasmonic screening. Conversely, excessive GQD coverage can preserve fluorescence but obstruct access to Au-associated recognition or electron-transfer sites.^68–70^ Similar trade-offs apply to ligand layers: increasing ligand density can improve chemical selectivity and colloidal stability while increasing tunneling distance and slowing charge communication. These competing effects imply the existence of an architectural operating window rather than a universally optimal composition.

A useful analytical framework is to distinguish between structural amplification and structural attenuation. Amplification occurs when nanoscale arrangement increases the fraction of recognition events that reach the readout channel; attenuation occurs when geometric separation, surface blockage, disorder, or parasitic relaxation dissipates that information before detection. The objective of design is therefore to maximize signal propagation while preserving chemically meaningful recognition.

This perspective also clarifies why composition alone is an incomplete descriptor of sensing performance. Two hybrids with identical nominal Au : GQD ratios may differ substantially if their interfacial contact area, surface accessibility, or degree of aggregation differs. Mechanistic interpretation should consequently prioritize descriptors that capture spatial organization and interfacial connectivity.^71–73^ Once architecture is treated as a mediator between recognition and transduction, performance evaluation can move beyond isolated metrics toward criteria that test whether the entire sensing pathway remains selective, interpretable, and robust under realistic perturbations.

Three evidential levels should be distinguished: a response change after hybridization, an analytical improvement over an appropriate comparator, and a non-additive contribution attributable to interfacial coupling. Establishing the second requires same-analyte comparisons with GQD-only and Au-only systems under matched recognition chemistry, component loading, and measurement conditions. A physical mixture or weakly coupled assembly can additionally help test whether interfacial organization is responsible for the improvement. Comparison with non-Au hybrids is needed before claiming an advantage specific to gold. Relevant endpoints include calibration slope, background variability, detection limit, interference tolerance, and reproducibility. For electrodes, accessible area and accumulation conditions must also be controlled. A larger fluorescence intensity or current alone establishes neither improved detection capability nor synergy.

Fig. 4 illustrates electrochemical changes during interface construction and target binding in a PSA immunosensor.^69^ Here, *E*_s_ denotes the screen-printed electrode; *E*_s_ alone identifies the bare substrate, whereas labels beginning with *E*_s_‖ indicate the corresponding modified configurations. This example illustrates architecture-dependent electrochemical transduction, and its antibody-mediated recognition cannot be directly extrapolated to metal-ion sensing. Panel (a) shows increasing oxidation peak current with increasing scan rate. This observation establishes scan-rate dependence but does not independently demonstrate diffusion control. Assigning a predominantly diffusion-controlled response would require analysis of background-corrected peak current (*i*_p_) against the square root of scan rate (*ν*_1/2_), with consideration of possible surface-associated and capacitive contributions. Since this relationship is not presented in Fig. 4, no definitive transport regime is assigned here. Panel (b) shows changes in the voltammetric response following sequential functionalization. The current does not decrease monotonically at every assembly step: PL addition produces a modest change, whereas subsequent antibody and BSA incorporation lowers the peak response, followed by a pronounced decrease after PSA binding. These observations are consistent with changes in electrochemical accessibility and interfacial blocking, although they do not uniquely establish the microscopic origin of each current change.

**Fig. 4:**
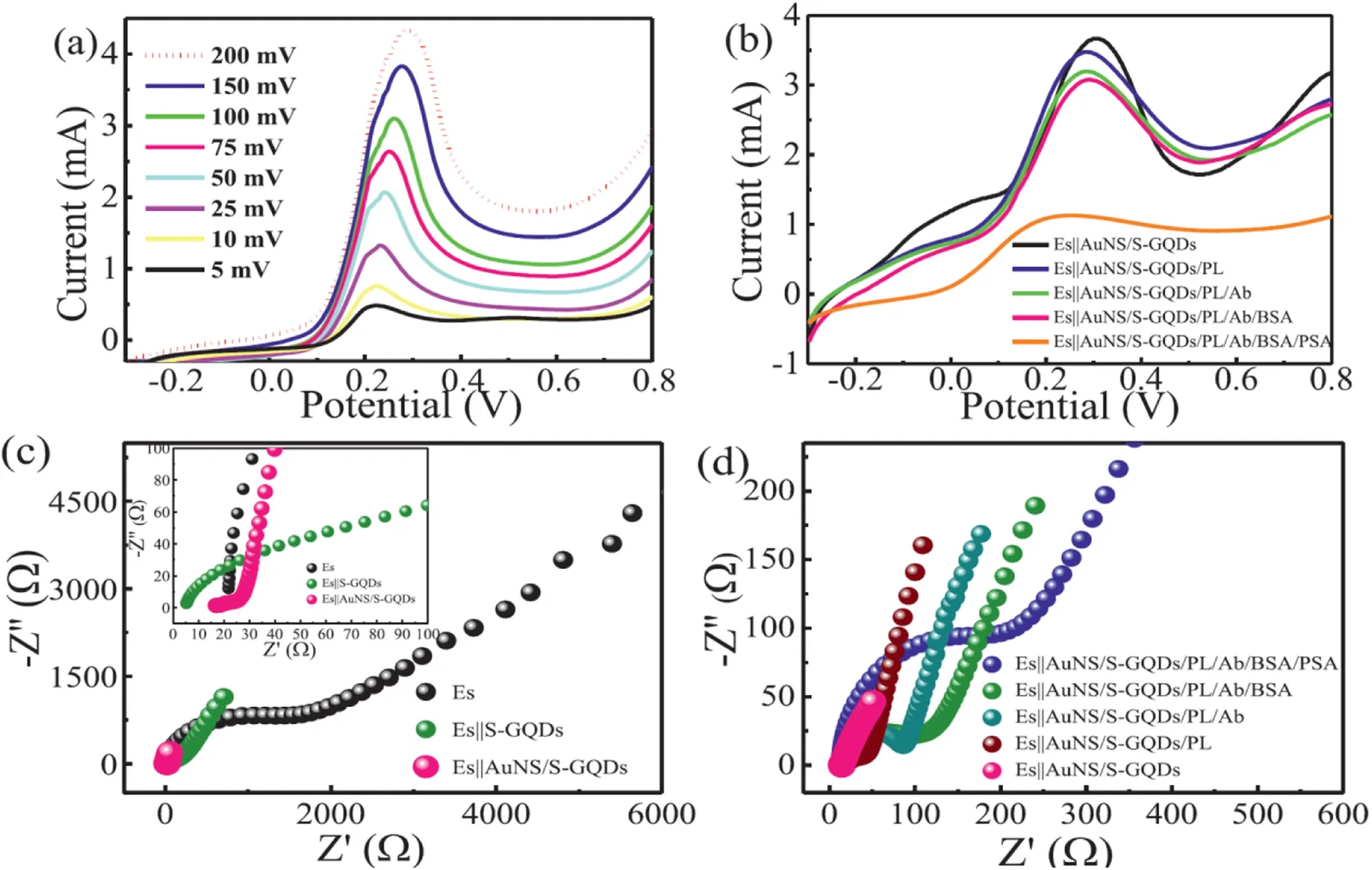
Electrochemical characterization of the PSA immunosensor: (a) scan-rate-dependent LSV, (b) sequential functionalization responses, (c) electrode-comparison Nyquist plots, and (d) Nyquist plots during biofunctionalization. *E*_s_ denotes the screen-printed electrode, with *E*_s_ alone indicating the bare substrate; AuNS, gold nanostars; S-GQDs, sulfur-doped graphene quantum dots; PL, poly-l-lysine; Ab, anti-PSA antibody; BSA, bovine serum albumin; PSA, prostate-specific antigen. Scan-rate units in panel (a) have been corrected to mV s^−1^. Adapted with permission from ref. 69. © 2021 Elsevier B.V.

Panel (c) reinforces this interpretation through EIS, where the marked decrease in charge-transfer resistance from the bare electrode to S-GQD- and AuNS/S-GQD-modified electrodes confirms that the hybrid architecture enhances interfacial conductivity rather than functioning as a passive support. Panel (d) then shows the reverse trend during biofunctionalization, with *R*_ct_ progressively increasing after addition of poly-l-lysine, antibody, BSA, and PSA, consistent with growing interfacial blocking. Critically, these results illustrate an architectural trade-off: the same nanostructure that amplifies electrochemical communication through increased active area and conductivity becomes progressively attenuated as selective recognition layers are assembled. Nevertheless, changes in current and impedance alone do not establish target specificity, because similar blocking effects may arise from nonspecific adsorption; therefore, these data are most appropriately interpreted as evidence of architecture-mediated signal propagation rather than as standalone proof of selective recognition.

### 3.4 Mechanism-based performance criteria and design trade-offs

A mechanistically consistent evaluation framework should assess whether recognition, transduction, and architectural coupling operate coherently rather than relying on a single analytical figure of merit. Sensitivity, for example, reflects the magnitude of signal change per change in analyte concentration, but it does not by itself establish chemical selectivity or mechanistic specificity. A steep response can originate from nonspecific aggregation or matrix-dependent surface restructuring. Similarly, a low detection threshold may be obtained under conditions where the active interface is unstable, difficult to regenerate, or highly sensitive to pH and ionic strength. Performance metrics therefore need to be interpreted in relation to the physical origin of the signal.

Operationally, the framework separates analytical response from mechanistic attribution. Sensing architecture should be examined by identifying the proposed recognition site, the analyte-induced interfacial change, the measured readout, and the controls that connect these steps. For fluorescence systems, spectral overlap, lifetime measurements, and absorption-corrected intensity data can help distinguish optical attenuation from changes in excited-state relaxation, although no single measurement necessarily resolves all alternatives. For electrochemical systems, comparisons with Au-only, GQD-only, and recognition-layer controls can help separate hybrid-mediated transduction from increased electrode area, nonspecific adsorption, or interfacial blocking. Where these controls are unavailable, the reported sensing response remains relevant, but its assignment to a specific Au–GQD coupling mechanism should be treated as provisional. This distinction provides a consistent basis for comparing platforms without equating a low detection limit with strong mechanistic evidence.

Selectivity is particularly dependent on the ratio between target-induced perturbation and background interfacial variability. The relevant design objective is not simply to strengthen target binding but to reduce the overlap between target and interferent response distributions. Stability should likewise be understood as preservation of the recognition–transduction relationship over time, rather than retention of signal intensity alone.^74–76^ Drift can arise from Au aggregation, GQD oxidation, ligand rearrangement, surface fouling, or progressive alteration of interfacial charge, each of which changes the mechanistic meaning of the readout.

Dynamic range introduces an additional trade-off. High-affinity recognition can increase responsiveness at low concentration but may lead to early site saturation, whereas weaker interactions can extend the working range at the cost of reduced low-concentration response. Reversibility presents a similar balance: strong coordination can improve capture efficiency while limiting regeneration. These tensions indicate that sensor optimization is intrinsically multi-objective.

A transferable design framework should therefore evaluate Au–GQD hybrids through four linked criteria: chemical differentiation at the recognition interface, efficient propagation of the binding perturbation, transduction specificity, and structural stability of the coupled interface. Optimization of one criterion at the expense of the others is unlikely to produce a robust analytical platform.^77–79^ This mechanistic framework establishes the basis for interpreting experimental evidence without conflating conceptual design principles with reported performance. The subsequent evidence-focused analysis can therefore examine how effectively published Au–GQD systems realize these design requirements in practice.

The schematic integrates the principal design layers governing Au–GQD sensing interfaces, from chemically differentiated recognition sites to measurable analytical output. Selective ion binding first perturbs the interfacial environment, which is translated through fluorescence or electrochemical pathways and further modulated by nanoscale architecture (Fig. 5). Au–GQD separation, surface coverage, ligand thickness, and connectivity determine whether this perturbation is amplified or attenuated. The resulting performance therefore reflects a balance among recognition specificity, transduction efficiency, structural organization, reversibility, stability, and signal robustness.

**Fig. 5:**
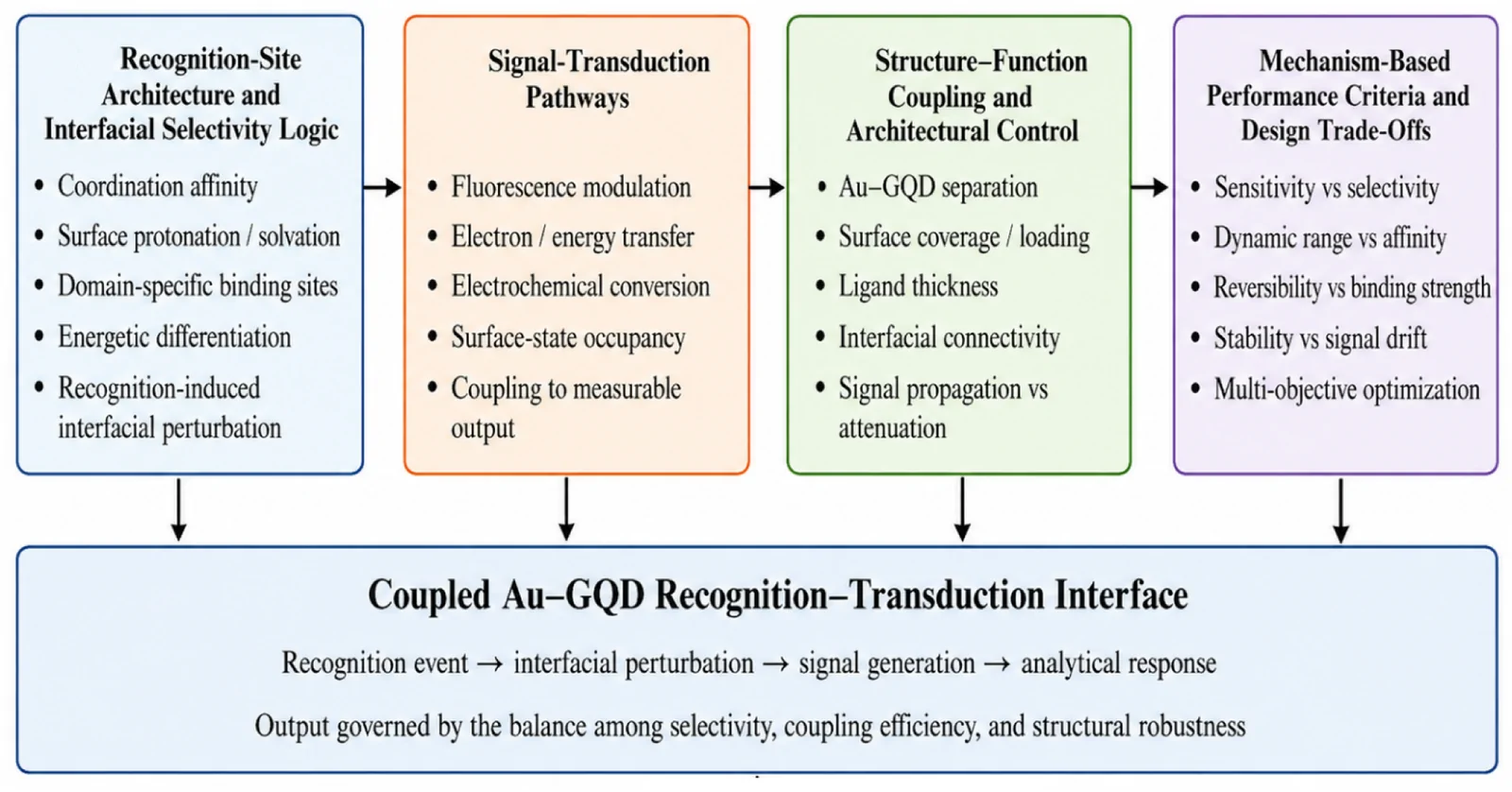
Mechanistic framework linking interfacial recognition, signal transduction, architectural control, and performance trade-offs in Au–GQD hybrids toward selective and robust analytical response.

## 4 Evidence and limitations of Au/GQD-based platforms for metal and metalloid sensing

The studies considered in this section represent distinct levels of evidence rather than a uniform class of coupled Au–GQD sensors. Preassembled AuNP/GQD interfaces, bimetallic cluster composites, supported sensing films, dual-emission nanomixtures, and reaction-generated Au reporters are therefore evaluated separately. Hg^2+^, Pb^2+^, Cu^2+^, Cd^2+^, and arsenic provide environmentally relevant analytical examples, whereas Cr^3+^ detection does not establish Cr(vi) sensing capability. Au^3+^ and Fe^3+^ studies are retained as additional metal-ion applications rather than representative demonstrations of priority toxic-pollutant monitoring. The available studies support architecture-specific observations and identify evidence gaps; they do not establish universal relationships between Au–GQD structure and analytical performance.

### 4.1 Fluorescence responses of AuNP/GQD interfaces and AuNC–GQD nanomixtures

Among the supplied studies, fluorescence modulation provides the clearest direct evidence for Au/GQD-based metal-ion ion sensing. The Cr^3+^-responsive AuNP/GQD system shows that the optical role of Au is highly dependent on nanoparticle concentration rather than being a fixed material attribute. At low AuNP concentration, the hybrid exhibited a 2.67-fold increase in GQD photoluminescence, whereas higher Au loading generated a new excitation feature at 421 nm and selectively enhanced particular radiative modes.^80^ This comparison supports an Au-associated increase in baseline GQD emission under the reported conditions, but does not establish a corresponding improvement in Cr^3+^ detection capability. Such a conclusion would require matched Cr^3+^ calibrations and background measurements for GQDs alone and the hybrid. Likewise, the optical enhancement does not demonstrate superiority over an Au-only sensor or a non-Au hybrid.

The Cr^3+^ study reports a concentration-dependent fluorescence response over 1–400 µM, a *R*^2^ = 0.9736, and an observable response at 50 ppb.^80^ These observations demonstrate analytical responsiveness but do not establish a statistically defined limit of detection (LOD). The source does not provide an explicit rationale for the absence of a numerical LOD in the reported sensing discussion. Its emphasis on plasmon-enhanced photophysics and demonstration of ion-responsive fluorescence provides context for the reporting scope, but should not be interpreted as the authors' stated reason for omitting this metric. Neither the lowest calibration concentration nor a detectable response at a selected concentration is equivalent to an LOD established using a defined statistical criterion.

Consequently, the Cr^3+^ platform cannot be ranked by detection capability against the Cu^2+^/Cd^2+^ ratiometric sensor, for which LODs of 4.12 × 10^−9^ M and 9.43 × 10^−7^ M, respectively, are reported.^81^ A calibration *R*^2^ describes association between concentration and response, whereas an LOD depends on the separation of low-concentration signals from background variability. The missing Cr^3+^ value therefore represents a reporting limitation, not evidence of intrinsically poorer detection capability. Comparison remains informative at the architectural level—plasmon-mediated modulation *versus* separate reference and responsive emission channels—but numerical performance ranking requires compatible LOD definitions, calibration procedures, and matrix conditions.

A more analytically differentiated strategy appears in the Cu^2+^/Cd^2+^ ratiometric system, where nitrogen-doped GQDs provide a stable emission at 403 nm while the gold-nanocluster emission at 565 nm responds oppositely to the two ions. Cu^2+^ quenches the AuNC channel, whereas Cd^2+^ enhances it, producing ion-dependent directional changes in the intensity ratio. The responsive component is glutathione-capped AuNCs, while N-GQDs provide the approximately invariant reference emission.^81^ Glutathione contains thiolate anchoring groups and accessible carboxylate and amino functionalities, providing a ligand-defined chemical environment around the AuNCs. The source attributes Cu^2+^-induced quenching to charge transfer and Cd^2+^-induced enhancement to formation of core–shell heterostructures.

These assignments place the recognition-related perturbation at the ligand-protected AuNC component rather than demonstrating direct recognition by N-GQDs. However, response direction alone does not identify the coordinating donor atoms, electron-transfer direction, shell composition, or binding stoichiometry. Establishing those details requires ligand-sensitive spectroscopy, structural or elemental characterization, and lifetime measurements. The reported detection limits of 4.12 × 10^−9^ M for Cu^2+^ and 9.43 × 10^−7^ M for Cd^2+^ demonstrate different analytical sensitivities within the same ratiometric architecture, but do not independently quantify binding affinity or establish simultaneous mixed-ion determination.^81^

Comparison therefore suggests two distinct fluorescence design philosophies: plasmon-mediated amplification of the GQD signal and ratiometric partitioning of reference and responsive channels. The latter provides a clearer route for compensating background intensity fluctuations, whereas the former emphasizes nanoscale optical coupling.^66,73^ Together, these studies indicate that fluorescence performance depends not simply on emission strength but on how the Au and GQD domains are assigned specific analytical roles.

Fig. 6 illustrates the Cr^3+^-dependent fluorescence response of the AuNP/GQD platform.^80^ Panels (a) and (b) compare individual metal-ion responses at the reported concentration of 50 µM. Cr^3+^ produces approximately 46% fluorescence suppression, whereas Cu^2+^ and Co^2+^ produce approximately 20–25% suppression. These responses indicate preferential attenuation by Cr^3+^ under the tested conditions, but also appreciable cross-response to competing ions. The figure therefore supports partial discrimination rather than high specificity. Individual-ion measurements do not establish whether Cr^3+^ can be quantified accurately in mixtures containing variable concentrations of Cu^2+^ or Co^2+.^

**Fig. 6:**
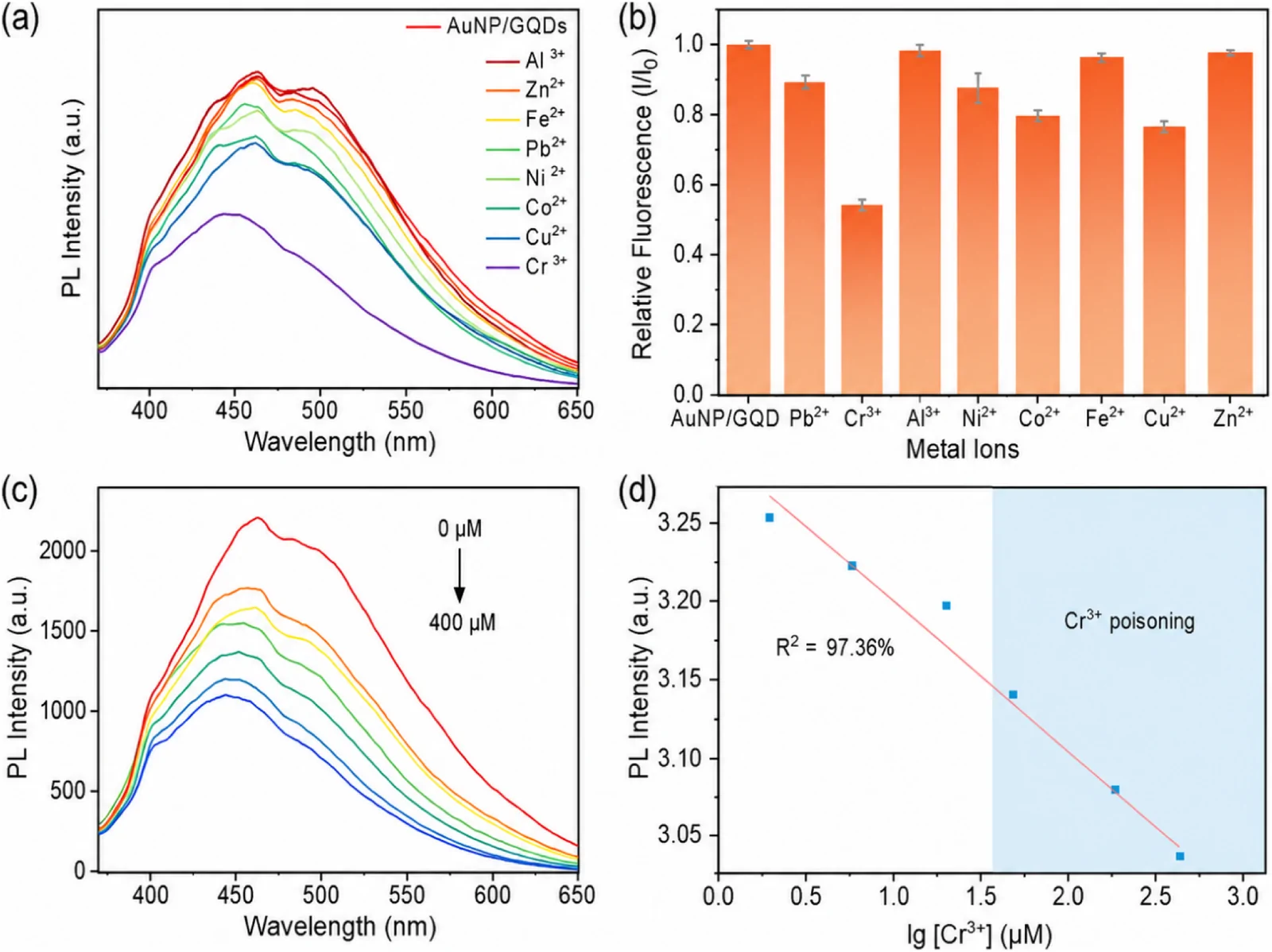
AuNP/GQD fluorescence responses: (a) metal-ion spectra, (b) individual-ion responses at 50 µM, (c) Cr^3+^ titration, and (d) calibration with *R*^2^ = 0.9736. Shading reproduces the source authors' poisoning annotation, not a validated clinical threshold. Reproduced with permission from ref. 80. © 2023 Chinese Laser Press.

Panel (c) shows decreasing fluorescence with increasing Cr^3+^ concentration, while panel (d) presents approximately six plotted calibration points against logarithmic concentration. The displayed *R*^2^ of 97.36% is a coefficient of determination, expressed conventionally as *R*^2^ = 0.9736, rather than a *R*^2^. It describes agreement with the fitted relationship but does not independently establish detection capability or predictive reliability. The limited calibration points require cautious interpretation, including consideration of residuals, replicate variability, and independent validation.

The shaded region labelled “Cr^3+^ poisoning” reproduces the source authors' proposed concentration-based interpretation. It is not an experimentally established clinical boundary in this review, nor evidence that the aqueous calibration remains valid in blood. Accordingly, the figure is interpreted as evidence of concentration-dependent fluorescence attenuation with incomplete selectivity and validation, rather than a clinically validated poisoning assay.

### 4.2 Electrochemical and supported-film detection of Hg^2+^, Pb^2+^, and arsenic

An AuNP–GQD electrode provides direct evidence for electrochemical detection of Hg^2+^ and Cu^2+^.^93^ The study employed GQD-functionalized, cysteamine-capped Au nanoparticles in an anodic stripping configuration and reported detection limits of 0.02 nM for Hg^2+^ and 0.05 nM for Cu^2+^, with corresponding signal-to-noise ratios of 6.25 and 4.81. These values should retain their reported definitions rather than being treated as equivalent to detection limits calculated using a common statistical criterion. Unlike fluorescence attenuation, stripping measurements depend on analyte accumulation, electrochemical deposition, and subsequent oxidation. Au-mediated accumulation and GQD-associated surface functionality may contribute jointly, but their individual contributions require matched component controls and characterization of the deposited analyte. The study establishes an assembled AuNP–GQD sensing architecture without, by itself, isolating an electronic-coupling advantage.

Pb^2+^ detection has also been demonstrated using an Au–Cu nanocluster/N-GQD composite, with a reported detection limit of 1 pM.^94^ The source assigns selectivity to interactions involving Au(i) sites and attributes improved electron transport and Pb^2+^ reduction to the electron-rich N-GQD component. These assignments should be distinguished from independently isolated causal contributions. Importantly, this is a bimetallic system: Cu incorporation changes the cluster composition and electronic structure, so its performance cannot be attributed exclusively to a binary Au–GQD interface. Comparison with Au-only, Cu-only, N-GQD-only, and physical-mixture electrodes under matched conditions is necessary to resolve the contributions of cluster composition and assembly.

Arsenic sensing extends the evidence to a supported-film architecture. An electrodeposited polypyrrole/GQD layer on gold-coated glass was used for SPR detection of arsenic reported as As(iii), over 0.005–10 ppm, with a stated sensor limit of 0.005 ppm.^95^ Au supplies the plasmonic film, while the overlying composite constitutes the sensing layer. Consequently, the measured response involves changes in the optical properties of the supported layer and does not independently demonstrate Au–GQD charge transfer. This example broadens analyte coverage but does not establish As(iii)/As(v) discrimination, nor should its stated sensor limit automatically be equated with a statistically validated detection limit.

### 4.3 Additional metal-ion applications and adjacent transduction examples

The Au^3+^ paper assay is classified separately because gold is initially the analyte and subsequently the reaction-generated optical reporter, rather than a pre-existing metallic sensor component.^82^ The extraction–reduction sequence provides a useful analytical example, but it does not validate recognition at a preassembled Au–GQD interface. Similarly, the Fe^3+^-responsive GQD–Au–Ni micromotor represents a multicomponent device whose signal cannot automatically be assigned to binary Au–GQD coupling.^83^ Au^3+^ and Fe^3+^ remain relevant metal-ion targets in specific applications, but these studies should not serve as representative evidence for monitoring conventional toxic-metal pollutants.

Several of the supplied studies do not target metal-ion ions directly, but they remain relevant because they reveal transferable transduction principles that can inform Au/GQD metal-ion sensor design. The Au–GQD surface plasmon resonance platform for phenol detection demonstrates that adding a GQD layer to a gold thin film can alter refractive-index sensitivity and interfacial optical response. The modified film achieved a detection limit of 0.1 µM compared with 1 µM for bare gold, together with a sensitivity of 0.02199° µM^−1^.^84^ For metal-ion sensing, the transferable lesson is not the phenol result itself but the evidence that GQD incorporation can measurably reshape the optical field and surface response of a gold substrate.

The AuNP/N-GQD/rGO hydrazine sensor^85^ provides an adjacent example of electrocatalytic signal generation, not a model of metal-ion recognition. Its reported performance concerns hydrazine oxidation and does not establish metal accumulation, deposition-potential selectivity, underpotential deposition, or Au–Hg amalgamation. Its relevance to the present review is therefore limited to the influence of hybrid electrode organization on electrochemical response; transfer to metal-ion stripping analysis would require independent accumulation and stripping experiments. A different transduction strategy is represented by the AuNP-quenched GQD aptasensor for phthalate esters.^86^ Target binding releases GQDs from the AuNP carrier, converting recognition-induced spatial separation into fluorescence recovery. The reported detection limit of approximately 4 pg mL^−1^ demonstrates the analytical leverage that can arise when nanoscale displacement directly controls quenching efficiency. Similarly, the GQD/AuNC ratiometric sulfide probe uses a stable GQD channel and a chemically responsive AuNC channel, achieving a 0.0064 µM detection limit and recoveries of 95.3–103.3%.^87^

These four studies should therefore be interpreted as adjacent mechanistic evidence rather than direct metal-ion sensing demonstrations. Collectively, they identify four transferable routes—SPR modulation, electrocatalytic amplification, distance-controlled fluorescence recovery, and dual-emission ratiometry—that can broaden the design space of Au/GQD metal-ion sensors without being misclassified as metal-ion detection evidence themselves. The present synthesis does not comprehensively cover aggregation-based AuNP colorimetry, DNAzyme- or aptamer-mediated metal-ion assays, or surface-enhanced Raman spectroscopy. These routes involve additional recognition and reporting elements that cannot be inferred from Au/GQD composition alone. They define complementary areas beyond the bounded evidence considered here; the conclusions therefore concern selected Au/GQD architectures rather than the full landscape of gold-based metal-ion sensing.

### 4.4 Architecture-specific observations and gaps in analyte coverage

The reviewed systems illustrate different functional assignments rather than establishing a universal Au–GQD synergistic advantage. In the Cr^3+^ platform, Au incorporation modifies GQD photoluminescence.^80^ In the Cu^2+^/Cd^2+^ system, N-GQDs provide a reference emission while AuNCs supply the responsive channel.^81^ In the Au^3+^ paper assay, GQDs participate in generating the metallic reporter.^82^ These observations support distinct component roles, but their analytical consequences require appropriate controls. In particular, brighter emission, dual-channel reporting, or reaction-generated color should not be treated as evidence that the hybrid outperforms both individual components and alternative non-Au architectures.

The expanded evidence distinguishes several analytical configurations rather than establishing a common structure–performance relationship. The Cr^3+^ study examines an AuNP/GQD optical platform,^80^ whereas the Cu^2+^/Cd^2+^ study uses a responsive AuNC channel and an N-GQD reference channel within an electrostatically associated nanomixture.^81^ The Hg^2+^/Cu^2+^ electrode provides an assembled AuNP–GQD stripping platform;^93^ the Pb^2+^ sensor introduces a bimetallic Au–Cu cluster composite;^94^ and the arsenic SPR sensor combines a gold film with a polymer/GQD sensing layer.^95^ These configurations support different mechanistic questions and should not be pooled as equivalent evidence of interfacial electronic coupling.

Numerical performance comparisons are likewise conditional. Detection limits obtained from fluorescence, stripping current, or other electrochemical responses depend on different accumulation procedures, calibration models, noise definitions, and measurement conditions. Adding further studies therefore improves coverage but does not establish that one architecture is intrinsically superior. The Au^3+^ reaction-generated reporter and Fe^3+^ micromotor are retained outside the principal toxic-pollutant comparison.^82,83^

Important coverage gaps remain. The reviewed evidence does not establish a general Au–GQD strategy for As(v) or Cr(vi), and responses to As(iii) or Cr^3+^ cannot be extrapolated to those oxidation states. Arsenic and chromium speciation must be treated explicitly because oxidation state and solution conditions alter the species available for recognition and electrochemical conversion. These gaps concern the verified evidence assembled in this review and should not be interpreted as proof that no relevant sensors exist elsewhere. Accordingly, the framework organizes architecture-specific observations and validation requirements rather than asserting experimentally established relationships across all toxic metals and metalloids.

Fig. 7 presents individual-ion responses and selected interference tests for the N-GQD–AuNC ratiometric sensor.^81^ Panel (a) shows that Cu^2+^ decreases the normalized emission ratio, (*I*_565_/*I*_403_)/(*I*_565_/*I*_403_)_0_, whereas Cd^2+^ increases it. Panels (b) and (c) indicate that the respective target responses are approximately retained in the presence of the listed coexisting ions at the tested concentrations. However, panel (b) does not show Cd^2+^ as an interferent in the Cu^2+^ assay, and panel (c) does not show Cu^2+^ as an interferent in the Cd^2+^ assay. These results therefore support separate target responses and condition-specific tolerance to selected interferents, rather than simultaneous Cu^2+^/Cd^2+^ determination.

**Fig. 7:**
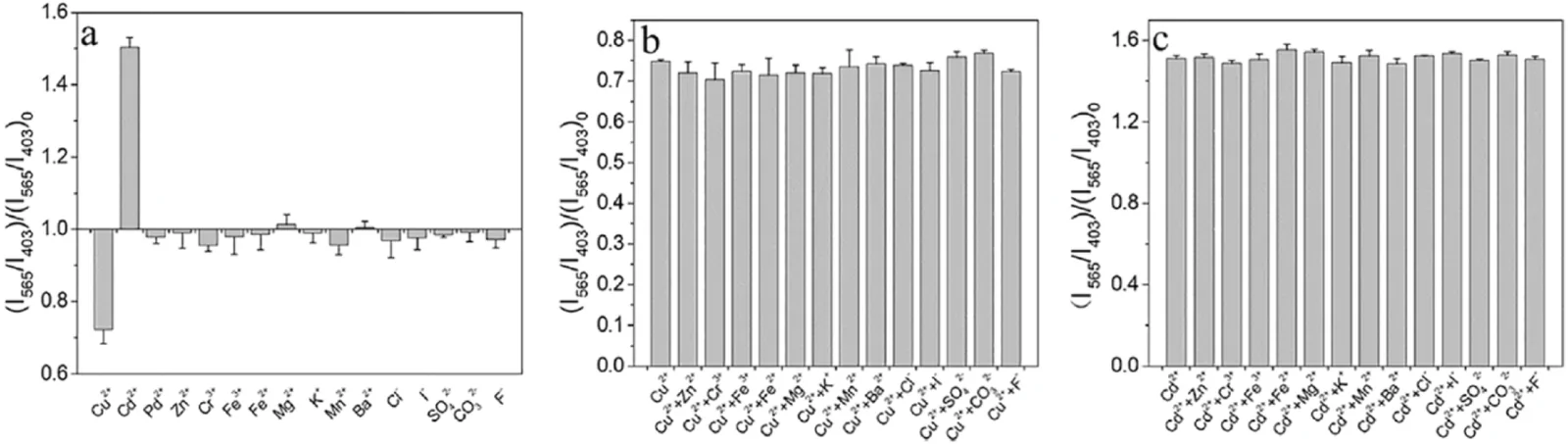
N-GQD–AuNC ratiometric responses to (a) individual ions, (b) Cu^2+^ with selected interferents, and (c) Cd^2+^ with selected interferents. Simultaneous Cu^2+^/Cd^2+^ quantification is not established. Adapted with permission from ref. 81. © 2022 Elsevier B.V.

This distinction is essential because both targets perturb the same AuNC-responsive emission at 565 nm, while the N-GQD emission at 403 nm serves as a nominally stable reference. In a mixture, their opposing effects may partially or completely compensate, potentially producing a near-baseline ratio despite the presence of both ions. The extent of compensation cannot be determined from the displayed single-target experiments, and additive response behavior should not be assumed without mixture measurements. A single ratio cannot uniquely determine two independently unknown concentrations without additional independent information. The reference channel does not supply a second target-dependent measurement merely because two emission bands are recorded. Reliable simultaneous analysis would require an independently responsive observable or validated selective separation or masking, followed by calibration and recovery testing across varying Cu^2+^ concentration ratios. Accordingly, the individual-target calibrations and detection limits reported in this study should not be extrapolated to simultaneous quantification in mixed samples.

Table 2 distinguishes optical modification, analytical improvement, and external benchmarking. Increased baseline fluorescence in the Cr^3+^ and Fe^3+^ platforms supports Au-associated changes in GQD emission, but does not independently establish improved metal-ion detection.^80,90^ A direct analytical comparison is provided by the phenol SPR platform, which reports a lower detection limit than the Au-only film; this advantage remains specific to its analyte and measurement conditions.^84^ For Cu^2+^, Au-free N-GQDs and a GQD/MOF architecture demonstrate alternative recognition and reporting strategies.^91,92^ Likewise, Au/GQD and Au-only stripping systems both enable sensitive Hg^2+^ detection, although their accumulation conditions and detection criteria differ.^88,93^ These comparisons identify relevant alternatives without assigning performance differences solely to Au–GQD coupling. A defensible claim of hybrid advantage requires matched target exposure, recognition chemistry, material loading, background characterization, and interference testing. The evidence therefore supports architecture-specific functions and selected improvements rather than universal Au/GQD superiority.

**Table 2:** Analytical performance and comparative evidence for Au/GQD sensing platforms, component-only systems, and non-Au alternatives

Platform/ref.	Analyte	Transduction and component roles	Analytical performance	Comparative evidence	Implication for hybrid advantage
AuNP/GQD^80^	Cr^3+^	Au-modulated GQD fluorescence followed by target-induced quenching	Response range: 1–400 µM; *R*^2^ = 97.36%; baseline emission enhancement: 2.67-fold	Hybrid emission compared with GQD-only emission	Demonstrates optical enhancement; improved Cr^3+^ detection requires matched calibration and background comparisons
N-GQD/GSH-AuNC^81^	Cu^2+^	Stable GQD reference at 403 nm; quenched AuNC channel at 565 nm	Range: 0.08–6 µM; LOD: 0.00412 µM; scallop analysis	Distinct reference and responsive channels; interference testing	Supports ratiometric detection; improvement over AuNC-only sensing requires matched precision and matrix evaluation
N-GQD/GSH-AuNC^81^	Cd^2+^	Stable GQD reference; enhanced AuNC emission	Range: 1–40 µM; LOD: 0.943 µM; scallop analysis	Opposite response to Cu^2+^; interference testing at selected conditions	Supports analyte-dependent signaling; concentration-dependent interference tolerance remains unresolved
GQD/PVC-coated paper^82^	Au^3+^	Membrane extraction followed by GQD-mediated reduction and AuNP color formation	Range: 200–1000 µM; LOD: 70 µM; water analysis	Results compared with ICP-OES	Supports analytical applicability; reaction-generated Au must be distinguished from a preassembled Au/GQD interface
GQD–Au–Ni micromotor^83^	Fe^3+^	GQD-containing micromotor with fluorometric, magnetic, and electrochemical functionality	Numerical range and LOD: —	Multicomponent architecture	Attribution requires separation of GQD, Au, Ni, and motion-dependent contributions
Au@N-GQD^90^	Fe^3+^	Coordination-associated fluorescence quenching and adsorption-dependent charge transfer	Subnanomolar detection reported; baseline fluorescence enhancement: >10-fold; serum and river-water analysis	Hybrid fluorescence compared with bare N-GQDs	Supports Au-associated optical modification; the gain in analytical performance requires same-analyte component benchmarking
GQD-functionalized AuNP electrode^93^	Hg^2+^/Cu^2+^	Electrochemical accumulation and anodic stripping	Reported detection limits: 0.02/0.05 nM; corresponding S/N: 6.25/4.81; sensitivities: 2.47/3.69 µA nM^−1^	Direct metal-ion stripping platform	Establishes relevant electroanalytical performance; accumulation conditions and detection criteria must accompany comparisons
Rotating Au electrode^88^	Hg^2+^	Accumulation followed by subtractive anodic stripping	Range: 0.2–400 nM; LOD: 0.05 nM in synthetic solution and 4 nM in urine; deposition times: 120 and 180 s	Au-only external benchmark for ref. 93	Demonstrates sensitive detection without GQDs; differences between studies do not isolate the GQD contribution
N-GQDs without Au^91^	Cu^2+^	Single-channel fluorescence sensing	Range: 0.1–10 µM; LOD: 0.06 µM	GQD-only external benchmark for ref. 81	Establishes an Au-free alternative; different material chemistry and measurement conditions prevent causal performance ranking
GQD/ZrBTB framework^92^	Cu^2+^	Photoluminescent sensing using immobilized GQDs	Cu-ion response demonstrated; numerical range and LOD: —	Pristine and immobilized GQD emission compared	Provides a non-Au hybrid alternative; support-dependent emission benefits do not constitute a matched comparison with Au/GQD
Au/GQD SPR film^84^	Phenol	GQD coating modifies the Au-film optical interface	Hybrid LOD: 0.1 µM; Au-only LOD: 1 µM; hybrid sensitivity: 0.02199° µM^−1^	Direct same-analyte comparison with Au-only film	Demonstrates improved detection over Au-only sensing for phenol; transfer to metal-ion recognition requires validation
AuNP/N-GQD/rGO electrode^85^	Hydrazine	Electrocatalytic oxidation at a ternary electrode	Range: 0.01–20 µM; LOD: 0.005 µM; sensitivity: 31 µA µM^−1^; recoveries: 98.0–104.0%	Optimized multicomponent electrode	Illustrates electrocatalytic response; does not establish metal-ion preconcentration or Au-specific recognition
AuNP-quenched GQD aptasensor^86^	Phthalate esters	Recognition-mediated GQD release and fluorescence recovery	Tested range: 0.001–50 ng mL^−1^; LOD: approximately 4 pg mL^−1^; selected-mixture recoveries: approximately 100%	Assembly characterization and non-target testing	Supports spatially controlled transduction; recognition originates from the assay-specific aptamer architecture
GQD/Al^3+^-assembled GSH-AuNC^87^	Sulfide	Stable GQD reference at 470 nm; quenched AuNC channel at 624 nm	Range: 0.02–200 µM; LOD: 0.0064 µM; recoveries: 95.3–103.3%; RSD: 2.3–3.4%	Separate reference and responsive channels	Supports sulfide-responsive ratiometry; Al^3+^ assists assembly and is not the quantified analyte
Au–Cu NC/N-GQD electrode^94^	Pb^2+^	Bimetallic cluster provides proposed recognition sites; N-GQD supports electron transport	Reported LOD: 1 pM	Binary Au–GQD attribution requires controls separating Au, Cu and N-GQD contributions	Relevant Pb^2+^ example; bimetallic architecture
PPy/GQD layer on Au film^95^	As(iii), as reported	Au film provides SPR transduction; PPy/GQD forms the sensing layer	Range: 0.005–10 ppm; stated sensor limit: 0.005 ppm	Does not independently establish Au–GQD charge transfer or As(iii)/As(v) discrimination	Supported-film sensing architecture

### 4.5 Critical appraisal of evidence quality and mechanistic interpretability

A critical reading of the available Au/GQD sensing literature reveals that the principal limitation of the evidence base is not a shortage of signal-generation concepts, but the unequal level of mechanistic and analytical substantiation supporting them. The studies considered here employ fluorescence enhancement, ratiometric emission, color development, surface-plasmon response, electrocatalytic current, target-induced fluorescence recovery, and multimodal detection. The reviewed examples employ different analytical configurations, including fluorescence modulation, ratiometric emission, anodic stripping, and surface plasmon resonance.^80,81,93,95^ Their reported detection limits should therefore be compared only after accounting for calibration procedures, blank variability, sample preparation, accumulation conditions, and matrix effects. Method-validation guidance provides the appropriate basis for evaluating these factors, rather than treating individual application papers as evidence of a universal benchmarking protocol.^97^

A second concern is the uneven depth of mechanistic attribution. Some studies associate the analytical signal with specific interfacial processes, such as Au-mediated modification of GQD emission, ion-dependent modulation of an AuNC channel relative to a stable GQD reference, or conversion of Au^3+^ to Au^0^ during color development.^81,82^ Nevertheless, the supplied evidence generally does not establish whether these proposed processes are uniquely responsible for the observed analytical response. Alternative contributions from aggregation, local dielectric changes, surface-state redistribution, altered adsorption, or matrix-induced restructuring are difficult to exclude from endpoint measurements alone. This distinction is important because a plausible mechanism is not equivalent to a mechanistically isolated one. The literature therefore supports several credible transduction interpretations, but the strength of causal assignment varies considerably between platforms.

Another weakness concerns the asymmetry between analytical optimization and mechanistic validation. Operating parameters such as component ratios, pH, deposition time, enrichment conditions, or material loading are optimized in several architectures,^80,85^ yet optimization identifies conditions that maximize response rather than necessarily explaining why those conditions are optimal. Without independent descriptors of interfacial state, an experimentally determined optimum can remain phenomenological. Similarly, strong recovery in real matrices demonstrates practical analytical compatibility under the reported conditions, but it does not by itself establish that the same recognition mechanism persists unchanged when matrix composition varies. Analytical robustness and mechanistic robustness should therefore be treated as related but non-identical attributes.

The evidence also exposes a broader classification problem. Au/GQD systems grouped under one material label may involve dispersed nanoparticles, nanoclusters, thin films, ternary carbon composites, paper-supported reaction layers, or micromotors.^81,84^ Composition-based grouping can consequently conceal differences in dimensionality, interfacial contact, transport regime, and signal origin. A more rigorous critical framework would distinguish systems according to the dominant analytical event—interfacial electronic modulation, emissive ratio change, spatial separation, catalytic conversion, refractive-index perturbation, or reaction-generated nanophase—before comparing performance.

Overall, the current literature establishes a diverse but mechanistically heterogeneous evidence base. Its main scientific weakness lies in insufficient alignment among material architecture, claimed recognition mechanism, and analytical readout, rather than simply in variations in sensitivity. This critical distinction provides a logical transition to Section 5, where the unresolved barriers to standardization, validation, and future development can be considered without repeating the evidence-level assessment presented here.

## 5 Current limitations, standardization challenges, and future research directions

### 5.1 Resolving mechanistic ambiguity at the Au–GQD recognition interface

A major barrier to the maturation of Au/GQD metal-ion sensors is the incomplete separation of recognition chemistry from signal-generation physics. In many architectures, the observed response can potentially arise from several concurrent processes, including coordination, electrostatic adsorption, surface-state perturbation, charge transfer, plasmonic coupling, aggregation, and local dielectric changes. The next stage of development therefore requires mechanistic studies designed to isolate causality rather than merely correlate signal variation with analyte concentration. Time-resolved fluorescence, potential-dependent spectroscopy, *operando* electrochemical methods, surface-sensitive characterization, and controlled spacer-distance experiments can be combined to distinguish excited-state processes from ground-state charge redistribution and structural reorganization. The objective should be to identify which interfacial event is necessary and sufficient for signal generation.

Mechanistic resolution is particularly important for differentiating genuine ion recognition from environmentally induced perturbation. Changes in pH, ionic strength, competing ligands, dissolved organic matter, and oxidation state can alter Au and GQD surface chemistry independently of the target ion.^80,83^ Future mechanistic frameworks should therefore include competitive binding thermodynamics, protonation equilibria, and redox speciation rather than treating metal concentration as the sole independent variable. Equally important is the distinction between adsorption and analytically productive recognition. A metal ion may bind strongly without causing a measurable signal if the binding site is electronically decoupled from the transduction domain.

An improved design strategy should therefore move toward experimentally validated recognition–transduction maps in which binding location, interfacial energy change, and signal pathway are resolved independently. Such maps would allow different Au/GQD configurations to be compared on a mechanistic basis and would reduce reliance on empirical optimization.

Clarifying these elementary processes is a prerequisite for defining meaningful performance descriptors, which in turn requires a more standardized framework for evaluating Au/GQD sensors.

### 5.2 Standardized performance metrics and benchmarking protocols

The absence of harmonized evaluation criteria remains a major obstacle to meaningful comparison among Au/GQD sensing platforms. Reported analytical figures of merit are strongly affected by calibration protocol, background subtraction, signal-processing method, sample matrix, interference design, and the statistical definition of detection limits. A future benchmarking framework should therefore specify not only detection limit and linear range but also sensitivity normalization, response time, selectivity against chemically relevant interferents, baseline drift, regeneration efficiency, batch-to-batch variability, and uncertainty in calibration parameters. The proposed benchmarking framework is informed by established guidance on analytical selectivity and single-laboratory method validation; its application to Au/GQD sensors is presented here as a recommendation, rather than as a protocol already validated across these platforms.^96,97^

Standardization is particularly necessary for hybrid platforms that employ different transduction modes. Fluorescence, electrochemical, plasmonic, and colorimetric outputs cannot be ranked directly unless performance is normalized against comparable recognition conditions and analyte exposure. For fluorescence systems, quantum yield, excitation stability, photobleaching, and inner-filter effects should be reported alongside analytical response. Electrochemical platforms require explicit control of active electrode area, charge-transfer resistance, background current, and enrichment time. For multimodal systems, each output channel should be independently validated before claims of synergistic performance are made.

Selectivity should be evaluated as the ability to determine the target analyte in the presence of other sample components under specified analytical conditions.^96^ Accordingly, interference testing should include chemically relevant competitors and realistic interferent-to-target concentration ratios, with the effects assessed through calibration changes, precision, recovery, and concentration bias.^97^ For Au/GQD platforms, particular attention should be given to species that perturb the same responsive channel or modify surface binding, aggregation, or redox behavior. These are proposed validation priorities for the reviewed architectures, rather than conclusions established by unrelated hydrazine or pharmaceutical assays.

A useful future objective is the development of reporting standards that define a minimum dataset for Au/GQD sensors, including structural characterization, recognition-site chemistry, calibration statistics, stability metrics, and matrix-dependent performance. Such standardization would make mechanistic and analytical comparisons more reproducible. Once performance can be evaluated consistently, the remaining challenge becomes whether the sensing architecture can retain these characteristics during fabrication, storage, and repeated operation.

### 5.3 Stability, manufacturability, and translation beyond laboratory-scale platforms

Practical translation of Au/GQD sensors requires greater attention to material stability and fabrication reproducibility. Au nanostructures can undergo aggregation, ligand exchange, and surface fouling, while GQDs may experience oxidation, photochemical alteration, or changes in protonation state during storage and use. Because the analytical response depends strongly on interfacial distance and surface chemistry, relatively small structural changes can shift calibration even when the bulk composition remains unchanged. Stability should therefore be assessed as preservation of the recognition–transduction relationship rather than simple retention of signal intensity.

Manufacturing introduces a second level of uncertainty. Batch variation in GQD size distribution, oxidation degree, heteroatom content, Au particle morphology, and ligand coverage can translate directly into variability in sensor performance. Future synthesis protocols should define quality-control descriptors that are measurable before sensor assembly. Spectroscopic signatures, particle-size distributions, zeta potential, surface elemental composition, and interfacial loading density could serve as acceptance criteria for reproducible production.^82,86^ For immobilized platforms, coating thickness, porosity, adhesion, and lateral uniformity should also be controlled because they determine transport and accessibility of recognition sites.

Scalability must be considered together with material consumption and process complexity. Architectures that require multiple purification, functionalization, or deposition steps may provide sophisticated signal behavior but become difficult to manufacture consistently. A rational translation strategy should therefore evaluate whether each added structural component contributes a measurable analytical function. Components that increase complexity without improving selectivity, robustness, or signal interpretability should be eliminated.

Long-term operation also requires systematic assessment of fouling, regeneration, shelf life, and calibration drift under realistic storage and environmental conditions. These factors determine whether a laboratory sensor can become a deployable analytical platform. After reproducible fabrication and operational stability are established, the next opportunity lies in designing sensing systems that extract more chemically specific information rather than merely generating stronger signals.

### 5.4 Next-generation Au/GQD platforms: multiparametric recognition and intelligent signal interpretation

The next generation of Au/GQD metal-ion sensors should move from single-output detection toward architectures capable of resolving chemically similar ions through multiparametric information. A single fluorescence intensity or current value often provides insufficient dimensionality to distinguish overlapping interfacial responses. Combining spectrally resolved fluorescence, lifetime changes, electrochemical impedance, potential shifts, or plasmonic response within one platform could create orthogonal signal dimensions, provided that each channel reports a distinct physical process.

This strategy would benefit from deliberately engineered spatial and chemical heterogeneity. Instead of producing one nominally uniform recognition surface, future Au/GQD systems could contain multiple controlled binding environments with different coordination preferences and transduction couplings. The resulting response pattern could function as a chemical fingerprint rather than a binary signal. Such designs require careful calibration because increasing signal dimensionality also increases the risk of cross-correlation and overfitting.

Data-driven analysis may become useful at this stage, particularly for deconvolving multichannel outputs. However, machine-learning integration should not substitute for mechanistic understanding. Models should be trained on independently characterized datasets with controlled variation in ion concentration, matrix composition, pH, and interfering species.^80,87^ External validation and uncertainty quantification are essential if predictive classification is to remain reliable outside the training domain. Physics-informed or mechanism-constrained models may be preferable to purely empirical classifiers because they can incorporate known relationships between interfacial chemistry and signal generation.

Integration with miniaturized electronics, microfluidics, and portable optical or electrochemical readers also offers a route toward automated monitoring. The design priority, however, should remain analytical traceability: each processing step from recognition to final classification must preserve a definable connection to the underlying chemical event. Thus, future progress is likely to depend less on maximizing one conventional metric and more on constructing reproducible, interpretable, and information-rich Au/GQD interfaces capable of distinguishing complex ion environments with quantified confidence.

## 6 Conclusion

Au/GQD hybrids offer a versatile materials platform for metal-ion ion sensing because the metallic, quantum-confined, and chemically functional components can be coupled across recognition and transduction length scales. The reviewed evidence indicates that nominal composition alone is insufficient to explain the observed responses. However, it does not establish a general synergistic advantage of Au/GQD hybrids over their individual components or non-Au alternatives; that conclusion requires matched, same-analyte comparisons. It is determined by how recognition sites, interfacial electronic states, nanoscale spacing, and the selected optical or electrochemical readout are structurally connected. The field currently contains several distinct sensing architectures, but their mechanistic comparability and analytical standardization remain limited.

Progress therefore requires a shift from empirical signal optimization toward explicit recognition–transduction design. Mechanistic validation should establish which interfacial event generates the observable signal, standardized protocols should enable cross-platform comparison, and manufacturing strategies should preserve the relevant interface during scale-up and prolonged operation. Future Au/GQD sensors are likely to become more effective when they move beyond single-channel responses toward multiparametric and mechanistically constrained platforms capable of resolving chemically similar ions under complex conditions. The most meaningful advance will therefore not be the pursuit of progressively lower detection limits in isolation, but the development of sensing systems in which structure; recognition chemistry, signal origin, analytical performance, and practical reliability are quantitatively connected within a unified design framework.

## Conflicts of interest

There are no conflicts to declare.

## Data Availability

No primary research results, software or code have been included and no new data were generated or analysed as part of this review.
